# Bacteriophage Therapy as a Promising Alternative for Antibiotic-Resistant *Enterococcus faecium*: Advances and Challenges

**DOI:** 10.3390/antibiotics13121120

**Published:** 2024-11-23

**Authors:** Laura Ribes-Martínez, Maria-Carmen Muñoz-Egea, Jose Yuste, Jaime Esteban, Meritxell García-Quintanilla

**Affiliations:** 1 Clinical Microbiology Department, IIS-Fundación Jiménez Díaz, Universidad Autónoma de Madrid, 28040 Madrid, Spain; lauraribes@outlook.com (L.R.-M.); mcmunozegea@fjd.es (M.-C.M.-E.); jesteban@fjd.es (J.E.); 2 CIBERINFEC-Consorcio Centro de Investigación Biomédica en Red (CIBER) de Enfermedades Infecciosas, 28029 Madrid, Spain; 3 MePRAM, Proyecto de Medicina de Precisión Contra las Resistencias Antimicrobianas, CIBER de Enfermedades Infecciosas (CIBERINFEC), Instituto de Salud Carlos III, 28029 Madrid, Spain; jyuste@isciii.es; 4 Spanish Pneumococcal Reference Laboratory, National Center for Microbiology, Instituto de Salud Carlos III, 28029 Madrid, Spain; 5 CIBERES-Consorcio Centro de Investigación Biomédica en Red (CIBER) de Enfermedades Respiratorias, Instituto de Salud Carlos III, 28029 Madrid, Spain

**Keywords:** *Enterococcus faecium*, vancomycin-resistant *Enterococcus* (VRE), bacteriophage, alternative therapy, phage therapy, antibiotic resistance, multidrug resistance, combination therapy

## Abstract

*Enterococcus faecium* is a Gram-positive bacterium increasingly identified as a critical nosocomial pathogen that poses significant treatment challenges due to its resistance to multiple antibiotics, particularly vancomycin-resistant *E. faecium* (VRE) strains. The urgent need for alternative therapeutic strategies has renewed interest in bacteriophage (phage) therapy, given phages specificity and bactericidal potential. This review explores the advancements in phage therapy against antibiotic-resistant *E. faecium*, including phage morphological diversity, genomic characteristics, and infection mechanisms. The efficacy of phage therapy in in vitro, ex vivo, and in vivo models and the compassionate use in clinical settings are evaluated, highlighting the promising outcomes of phage–antibiotic synergies and biofilm disruption. Key challenges and future research directions are discussed, with a focus on improving therapeutic efficacy and overcoming bacterial resistance. This review emphasizes the potential of phage therapy as a viable solution for managing multidrug-resistant *E. faecium* infections and underscores the importance of future investigations to enhance clinical applications.

## 1. Introduction

*Enterococcus faecium* is a Gram-positive bacterium classified as an ESKAPE pathogen, frequently isolated from a wide range of environments, including the gastrointestinal tracts of humans and animals, as well as in natural biomes such as water, soil, and plants [1]. While some *E. faecium* strains have demonstrated potential probiotic properties and can also be beneficial in the food technology industry [2,3], this bacterium has emerged as a significant nosocomial pathogen, particularly in healthcare settings [4].

*Enterococcus faecalis* has long been the predominant cause of enterococcal infections. However, since the 1980s, while the distribution varies between countries, the incidence of *E. faecium* has surged, now accounting for approximately 50% of enterococcal infections [5,6,7,8,9]. This significant shift is primarily due to the widespread emergence of vancomycin-resistant *E. faecium* (VRE) strains [10]. These strains have acquired specific genetic traits that enable them to thrive in hospital environments [11] where they act as opportunistic pathogens, particularly in immunocompromised individuals, including those undergoing chemotherapy, organ transplants, or invasive procedures are at high risk, as are patients with chronic illnesses such as diabetes or kidney disease. Furthermore, critically ill patients in intensive care units, on mechanical ventilation, or with prolonged hospital stays face a heightened risk. VRE infections can lead to severe conditions such as bloodstream infections (e.g., bacteriemia and infective endocarditis), urinary tract infections, intra-abdominal and pelvic infections, post-surgery wounds, and device-associated infections involving catheters and other implants [12,13,14].

Infections caused by *E. faecium* are especially challenging due to the organism’s inherent resistance to multiple antibiotics and its rapid ability to develop additional resistance mechanisms [15]. In particular, VRE infections are linked to increased mortality, higher healthcare costs, and prolonged hospital stays [16,17]. Moreover, VRE infections account for over 82% of healthcare-associated infections caused by *E. faecium* in the USA, 31% in South America, and more than 22% in Asia, with a prevalence of 17.2% in Europe and 26.8% in Africa. Mortality rates associated with these infections are concerning, with an in-hospital mortality rate of 18%, and rates exceeding 57% at 30 days and 69% at 90 days [18]. Globally, *E. faecium* infections are responsible for approximately 200,000 deaths per year [19].

Treatment typically involves combinations of cell wall-active agents, such as ampicillin and vancomycin, with aminoglycosides. Although newer antibiotics like daptomycin, linezolid, and tigecycline have been employed to combat resistant strains, resistance to these agents has also emerged [20,21]. Furthermore, the ability of *E. faecium* to form biofilms exacerbates treatment difficulties by creating reservoirs of persistent bacteria, which contribute to resistance and heighten the risk of recurrent infections [22,23]. Although pan-resistant strains of *E. faecium* have not been widely reported, the presence of multidrug-resistant isolates underscores the urgent need for alternative therapeutic options [24].

One promising therapeutic alternative is bacteriophage therapy, which has gained renewed attention as a potential solution to the escalating crisis of antibiotic resistance [25]. Bacteriophages, or phages, are viruses that specifically target and lyse bacteria after injecting their genetic material into bacterial cells. The life cycle of bacteriophages typically follows two main pathways: the lytic cycle, which culminates in bacterial destruction, and the lysogenic cycle, where the phage genome integrates into the host DNA. Alternatively, phages can follow the pseudolysogenic cycle, an intermediate state where the phage genome remains inactive in the cell without integrating or initiating lysis [26].

For therapeutic purposes, lytic phages are preferred due to their ability to rapidly lyse bacteria. Upon attaching to a bacterial surface receptor, phages inject their genome, which takes over the host machinery to obtain new phage particles. As these accumulate, they produce enzymes that degrade the bacterial cell wall, causing cell lysis and the release of progeny phages to infect neighboring cells [27]. Unlike broad-spectrum antibiotics, phages are highly specific, targeting only particular bacterial species or strains, which minimizes collateral damage to the beneficial microbiota and reduces the risk of fostering resistance [28,29].

Phage therapy is not a new concept. It was first discovered in the early 20th century and used to treat bacterial infections before the advent of antibiotics [30]. However, with the growing prevalence of multidrug-resistant bacteria, phage therapy has re-emerged as a promising approach [31]. Recent studies have described the effectiveness of phages against a variety of antibiotic-resistant pathogens, including *Staphylococcus aureus*, *Pseudomonas aeruginosa*, and *Klebsiella pneumoniae*, in both in vitro and in vivo models [32,33,34]. Phage therapy has also shown to be promising in compassionate use cases against various multidrug-resistant pathogens, including *Acinetobacter baumannii*, *Mycobacterium abscessus*, and *Pseudomonas aeruginosa* [35,36].

Research on phage therapy specifically targeting *E. faecium* is limited compared to other pathogens. A literature search on PubMed in August 2024 using the terms ‘phage therapy’ AND ‘*Enterococcus faecium*’ reveals approximately 54 studies on phage therapy for *E. faecium*, most of them (81.48%) from the last 5 years, in stark contrast to around 313 studies found with ‘phage therapy’ AND ‘*Klebsiella pneumoniae*’ and 643 for ‘phage therapy’ AND ‘*Pseudomonas aeruginosa*’. This gap is especially relevant considering the rising clinical significance of *E. faecium*. Moreover, the exploration of bacteriophages as a therapeutic option against this pathogen is relatively recent, having the earliest in vivo study published in 2002 [37]. This lack of research, coupled with the recent emergence of *E. faecium* as a significant nosocomial pathogen, underscores the urgent need for a comprehensive and up-to-date review of phage therapy against this organism.

In this review, we will describe the advances in phage therapy against *E. faecium*, encompassing both in vitro and in vivo studies. We will explore the potential of phages to combat antibiotic-resistant *E. faecium* infections and discuss the challenges and opportunities in translating this approach to clinical practice.

## 2. Diversity of Phages Targeting *E. faecium*

Research has uncovered a substantial diversity of phages with lytic activity against *E. faecium*, spanning morphology, genomes, host range, and infectivity. This diversity is essential for advancing phage therapy, as it allows for the careful selection of phages with the most appropriate characteristics for targeting specific infections.

### 2.1. Morphological Characteristics of E. faecium Phages

Phage classification is currently experiencing a major shift, now relying primarily on genomic techniques and methods. Previously, classification depended mainly on morphology [38,39]. Siphovirus morfotype—previously classified as the *Siphoviridae* family—is among the most frequently identified phages that infect *E. faecium*. These phages are characterized by their icosahedral heads and long, non-contractile tails. Examples include IME-EFm5 [40], vB_GEC_EfS_9 [41], IME-EFm1 [42], vB-EfmS-S2 [43], 9181, 9183, 9184 [44], vB_EfaS_HEf13 [45], vB_EfaS-271 [46], SSsP-1 [47], and vB_EfaS-Zip (Zip) [48]. Notably, siphovirus often exhibit broad lytic capabilities, which enhance their potential in clinical applications, particularly for addressing multidrug-resistant bacterial infections [49]. Podovirus phages—former *Podoviridae* family—are distinct due to their icosahedral heads and short, non-contractile tails. Representative examples include vB_EfaP-Max (Max) [48] and MDA1 [50]. Despite their simpler structural configuration, podovirus display significant lytic potential.

Members of the former *Myoviridae* family, now myovirus, are known for their icosahedral heads and contractile tails, which enhance their ability to penetrate bacterial cell walls. Phages such as EfV12-phi1—also previously known as Φ1—[51,52], MDA2 [53], Porthos [54], and iF6 [55] are of particular interest due to their potent lytic activity, which makes them effective even against vancomycin-resistant *E. faecium* strains. The contractile tails of these phages offer a mechanical advantage in breaking through bacterial defenses [56]. In the *Herelleviridae* family, phages share morphological similarities with myovirus, including icosahedral heads and contractile tails. A notable example is GVEsP-1 [47]. Although less extensively studied than other families, phages from *Herelleviridae* could offer additional therapeutic potential due to their lytic properties and structural versatility. 

### 2.2. Host Range Spectrum and Specificity

The host range of a phage, defined as the variety of bacterial strains it can infect and lyse, is a critical factor to consider in phage therapy. Phages that target *E. faecium* exhibit a remarkable diversity in their host ranges, which has significant implications for their therapeutic applications.

#### 2.2.1. Broad Host Range

Some phages exhibit a broad host range, allowing them to infect and lyse a high percentage of *E. faecium* clinical strains, including those resistant to multiple antibiotics. For example, Rigvava et al. showed that phage vB_GEC_EfS_9 had the ability to lyse 84% (59 out of 70) of *E. faecium* clinical isolates, including vancomycin-resistant strains [41]. Similarly, phage IME-EFm1 lysed 77.3% of the tested clinical *E. faecium* strains, including two vancomycin-resistant isolates [42]. Goodarzi et al. reported that phage vB-EfmS-S2 also exhibited a broad host range, lysing 82.3% among the 34 VRE clinical isolates tested [43]. In a recent study by Khazani Asforooshani et al., phage EF-M80 showed lytic activity against 60% of the tested clinical isolates, although it did not exhibit activity against other bacterial species [57]. The phage vB_Efs8_KEN04 [58], initially characterized for its efficacy against *E. faecalis*, highlights the potential benefits of polyvalent phages, as it has additionally shown activity against an *E. faeicum* strain and a potential broader host range has been hypothesized through predictive genomics.

Phages with a broad spectrum of activity are particularly attractive for phage therapy, as they can target a wider array of strains, potentially reducing the need for complex phage cocktails [59]. However, the broad targeting capacity of these phages raises concerns about collateral effects not only on commensal *E. faecium* strains but also on other members of the gut microbiota.

Certain broad-spectrum phages may have unintended off-target effects due to imperfect host specificity or the presence of homologous receptors in other bacterial species. This can result in the lysis of commensal bacteria, disrupting the broader microbiome. Such disruptions may diminish microbiome diversity and lead to adverse effects, such as loss of colonization resistance against pathogens (e.g., *Clostridioides difficile*), metabolic imbalances, or immune dysregulation. For example, in the gut, the depletion of commensals like *Bacteroides* spp. and *Lactobacillus* spp., which play roles in maintaining gut homeostasis and modulating immune responses, could exacerbate the risk of inflammatory conditions [60,61,62].

To address these concerns, broad-spectrum phages should be thoroughly screened for host range specificity using advanced techniques such as comparative genomics and host receptor analysis. The design of phage cocktails can also be optimized to preferentially target pathogenic *E. faecium* while sparing key commensal species. Additionally, post-therapy microbiota restoration strategies, such as probiotics or fecal microbiota transplantation, may mitigate the impact of phage-induced microbiota disruption.

#### 2.2.2. Narrow Host Range

On the other hand, some phages have a narrow host range, infecting only a specific subset of *E. faecium* strains. For instance, Raza et al. found that phage STH1 was only capable of lysing the vancomycin-resistant *E. faecium* strain used for its isolation, though it showed limited activity against two additional strains [63]. Similarly, phage IME-EFm5 was found to have a limited host range, making it less suitable for direct phage therapy. However, a promising alternative therapeutic agent was determined through its endolysin LysEFm5 [40]. Another study by Qu et al. demonstrated that the phage vB_Efm_LG62 (LG62) also showed a narrow host range, lysing only two out of the twelve tested *E. faecium* strains [64]. Although monovalent phages may limit the applicability of an individual phage, they can also offer certain advantages. For instance, a highly specific phage may minimize disruption to the host’s commensal microbiota, reducing the risk of dysbiosis [26,65]. Alternatively, phage directed evolution techniques, such as the Appelmans method, can be applied to these phages to extend their infective targets if necessary [54].

### 2.3. Genomic Analysis of Phages Infecting E. faecium

The genomic analysis of bacteriophages infecting *E. faecium* is critical for selecting appropriate candidates for therapeutic applications. By examining key genomic features, including genome size, open reading frames (ORFs), and the presence or absence of virulence or antibiotic resistance genes, it is possible to assess the safety and effectiveness of these phages for clinical use. Phylogenetic analysis, based on whole-genome sequencing and the comparison of specific genes, provides valuable insights into the evolutionary relationships between phages and their classification. Moreover, the genomic comparison of these phages allows for the identification of conserved and divergent genomic regions, which could be exploited to optimize the selection of phages with desirable therapeutic properties [66].

Phages that infect *E. faecium* display a broad spectrum of genome sizes, reflecting their genetic diversity. Phages such as IME-EFm5 and EF-M80 have genome sizes of approximately 40 to 42 kilobases (kb), while larger phages like GVEsP-1 can reach up to 149 kb. These genomic differences may influence the phages’ life cycles, host ranges, and therapeutic potentials [67]. ORFs identified within these phages are essential for various biological functions, including replication, structural assembly, and bacterial cell lysis. For instance, phage IME-EFm5 contains ORF31 that encodes the lytic enzyme LysEFm5, which has shown potential in degrading *E. faecium* cell walls. Similarly, other phages contain ORFs encoding proteins that play pivotal roles in essential phage functions such as DNA replication, packaging, morphogenesis, and cell lysis according to Wang et al.’s findings with 67 ORFs described for phage IME-EFm1 [42].

Moreover, one of the main concerns in the use of phages for therapeutic purposes is ensuring the absence of genes that could enhance bacterial virulence or contribute to the spread of antibiotic resistance. The presence of such genes in a phage genome could pose a significant risk if transferred to bacterial hosts. For example, phages IME-EFm1 and Ec-ZZ2 [68]—which have high genome homology—have been found to harbor a metallo-β-lactamase gene, raising concerns about its safety for therapeutic use due to the potential for horizontal gene transfer. These phages could be used only after genetic manipulation to eliminate undesirable genes. In contrast, phages such as EF-M80, LG62, Max, Zip, or neither of the phages recently studied by Soro et al. have undergone thorough genomic analysis, revealing no genes associated with virulence, lysogeny, or antibiotic resistance [69]. This absence of harmful genetic elements makes these phages more promising candidates for clinical applications [70].

On the other hand, certain phages possess genetic elements that enable them to bypass bacterial defense systems. The presence of anti-CRISPR loci in a phage genome can significantly enhance its efficacy by allowing the phage to evade bacterial resistance mechanisms. For instance, phage SSsP-1 has been found to contain potential anti-CRISPR loci, which may improve its ability to infect *E. faecium* strains that have evolved CRISPR-based defenses. This capacity to overcome bacterial resistance is particularly advantageous in the therapeutic context, where phages must remain effective against evolving bacterial populations. Beamud et al. and Mayo-Muñoz et al. have recently reviewed this matter extensively [71,72].

## 3. Phage Efficacy Against *E. faecium*: In Vitro Studies

### 3.1. Plaque and Liquid Assays

Both plaque and liquid media assays are essential tools for assessing the lytic activity of bacteriophages against *E. faecium*. These methods provide insight into the efficacy of various phages in reducing bacterial viability, with each approach offering unique advantages and revealing critical aspects of phage behavior.

Plaque assays provide a direct visual assessment of phage-induced bacterial lysis through the formation of clear zones (lysis halos). These halos are indicative of successful phage infection and subsequent bacterial lysis. For instance, the phage IME-EFm5 has been shown to produce distinct lysis halos on the *E. faecium* strain 4P-SA. Similarly, the phages Athos, Porthos, Aramis, d’Artagnan, and Planchet displayed robust lytic activity against various clinical isolates of *E. faecium*, as evident by the formation of clear plaques in double-layer agar assays. Plaque assays are also instrumental in determining the host range of phages, which is crucial for assessing their therapeutic potential. As previously mentioned, the phage vB_GEC_EfS_9 exhibited a broad host range lytic activity against 84% of the *E. faecium* strains tested. Other phages, such as S2 and vB_Efs8_KEN04, showed even broader activity, lysing 82.3% and 100% of clinical *E. faecium* isolates, respectively.

In contrast to plaque assays, liquid assays allow for a quantitative evaluation of bacterial reduction in the presence of phages, often through colony-forming unit (CFU) counts or optical density (OD) measurements. For instance, phage iF6 showed potent lytic activity against *E. faecium* FS86, inhibiting bacterial growth in both the exponential and stationary phases. Another example is phage vB_EfaH_163, which showed a significant reduction in viable cell counts at the 5 and 7 h marks across all MOI levels tested, but was inefficient in inhibiting bacterial proliferation after 24 h [73]. Moreover, these methods provide more detailed information about the kinetics of phage activity in environments that may mimic more accurately clinical conditions.

Liquid assays are particularly useful for evaluating phage–antibiotic synergy, which has been shown to enhance the efficacy of both treatments. Furthermore, liquid assays provide insights into phage kinetics, such as the latency period and burst size, which are critical for understanding the dynamics of phage infection.

### 3.2. Biofilm Disruption

Biofilms are communities of bacteria encased in an extracellular matrix and pose a significant barrier to effective antibiotic treatment, making infections difficult to manage. The unique ability of bacteriophages to penetrate and disrupt these biofilm structures offers a promising strategy for addressing chronic and recurrent infections caused by *E. faecium*. For example, research has described that phage vB-EfmS-S2 exhibits remarkable capability in inhibiting biofilm formation over periods ranging from one to five days, as well as degrading pre-existing biofilms within one to seven days [43].

The efficacy of phage S2 was assessed by measuring the reduction in colony-forming units (CFU), revealing its potential as a therapeutic agent against *E. faecium* biofilms. Another notable phage, vB_Efs8_KEN04, has also shown effectiveness in disrupting biofilms of *E. faecium*, particularly in multidrug resistant (MDR) clinical isolates. This phage activity was evaluated through biofilm formation, inhibition, and disruption assays, confirming its therapeutic potential. Another example is phage LG62, which has a narrow host range (2/12 strains), but inhibited biofilm formation by 30% [64].

On the other hand, phage 113 (ATCC 19950-B1), in combination with daptomycin (DAP) and other antibiotics (ampicillin, ciprofloxacin, and ertapenem), has been studied for its effectiveness in eradicating biofilms formed by the DAP-resistant strain R497 of *E. faecium*. Furthermore, phage cocktails comprising multiple phages have shown potential in combating biofilms. A cocktail containing phages 113, NV-497, NV-503-01, and NV-503-02, along with DAP and ampicillin, achieved significant elimination of biofilms formed by MDR *E. faecium* strains. Phages NV-497, NV-503-01, and NV-503-02 have individually and collectively exhibited activity against *E. faecium* biofilms. The ability of these phages to penetrate and degrade biofilms is crucial for the management of chronic infections. Biofilms create a protective environment for bacteria, shielding them from both antibiotics and the host immune system, which leads to persistent and recurrent infections.

Table 1 compiles key studies showcasing the effectiveness of individual phages and phage cocktails in disrupting biofilms.

### 3.3. Synergistic Effects of Phage Cocktails and Antibiotics

The application of phage cocktails in therapeutic contexts has garnered increasing attention due to their potential to enhance the spectrum of bacterial eradication and mitigate the development of resistance. The underlying principle of this approach is that combining multiple bacteriophages can overcome the limitations often encountered with monophage therapy. Resistance to phages remains a significant challenge in bacteriophage therapy, and the deployment of phage cocktails offers a promising strategy to counter this issue by providing multiple, simultaneous mechanisms of attack against bacterial targets.

Achieving a broader host range is critical, particularly when addressing the genetic diversity inherent in *E. faecium*. Targeting a diverse set of bacterial strains is essential for effective therapeutic coverage. For instance, one notable example of such synergy was the already mentioned phage cocktail composed of 113, NV-497, NV-503-01, and NV-503-02, when used in combination with the antibiotics daptomycin (DAP) and ampicillin (AMP), exhibited significant efficacy in eradicating biofilms formed by multidrug-resistant (MDR) strains of *E. faecium*. In another study, phage 113, combined with DAP and various β-lactam antibiotics such as AMP, cefotaxime (CPT), and ertapenem (ERT) exhibited remarkable synergy and bactericidal activity against the DAP-resistant *E. faecium* strain R497. This combination not only eradicated the bacterial population but also prevented the emergence of further resistance. Likewise, combining phage 113 with DAP and β-lactam antibiotics (CPT or ERT) proved effective against the *E. faecium* strain HOU503. Additionally, the same phage cocktail (113, NV-497, NV-503-01) used alongside DAP and AMP resulted in significant bacterial elimination in biofilm-associated infections. Similarly, the phage cocktail described by Wandro et al. [79], combining the use of phages Ben, Bop, and V12, resulted in a synergistic suppression of bacterial growth over a period of 72 h in strains of *E. faecium* and *E. faecalis*. These findings underscore the capacity of phage cocktails to enhance therapeutic outcomes by broadening the range of susceptible bacterial strains.

As has been shown, beyond the use of phage cocktails alone, combining phages with antibiotics can produce synergistic effects that significantly improve therapeutic outcomes. This synergy refers to an interaction where the combined effect of phages and antibiotics surpasses the sum of their individual effects. Various mechanisms may contribute to this synergy. Lusiak-Szelachowska et al. suggested six mechanisms to explain the phenomenon of phage-antibiotic synergy: (1) cell elongation/filamentation by antibiotics; (2) increased plaque size by antibiotics, accelerated phage amplification and enhanced burst size; (3) decrease in phage and/or antibiotic-resistant mutant appearance; (4) increased antibiotic susceptibility due to the presence of the phage; (5) lowered MIC of antibiotics after adding phages to an antibiotic; and (6) depolymerization of the bacterial polysaccharides by phage enzymes (glycan depolymerases) that increase antibiotic diffusion and cell penetration [81,82,83].

However, the interaction between phages and antibiotics is not always straightforward. For instance, the combination of phage NV-497 with DAP or CPT exhibited clear synergy in treating the *E. faecium* strains R497 and HOU503. Yet, when combined with linezolid (LNZ), an antagonist effect was observed, reducing the overall efficacy. Similar results were seen with phage NV-503-01, where synergy was evident with DAP and CPT, but antagonism emerged when combined with LNZ or minocycline (MIN).

These findings emphasize that while the potential for synergy between phages and antibiotics is considerable, not all combinations are beneficial. Some antibiotics, particularly those that inhibit protein synthesis, may interfere with phage replication thus reducing the overall therapeutic effectiveness.

Recent research has shed light on the complex mechanisms underpinning the interaction between phages, antibiotics, and bacterial resistance. For example, the study by Morrisette et al. revealed that combining phage 113 with DAP effectively prevents the development of resistance to phage 9184. This result suggests that phage cocktails not only expand the host range but may also help in combatting phage resistance, a crucial consideration for long-term phage therapy. However, the efficacy of phage cocktails is not universally consistent across all bacterial strains. In a previous study by Morrisette et al., the addition of phages to antibiotic therapy did not always enhance bacterial eradication, highlighting the importance of strain-specific phage selection. This variability suggests that a personalized approach, tailoring the selection of phages to the specific bacterial strains present in each infection, may be necessary to maximize therapeutic success [77].

Another intriguing area of research involves the impact of bacterial mutations on phage resistance and antibiotic susceptibility. Studies by Canfield et al. investigated phages 9181, 9183, and 9184 and revealed a phenomenon where bacterial mutations that confer resistance to phages also increase susceptibility to specific antibiotics. For instance, mutations in the *sagA* gene, which encodes a cell wall hydrolase, granted resistance to phage 9181 but concurrently heightened susceptibility to ampicillin and ceftriaxone. Similarly, mutations in the *epaR* and *epaX* genes, which are involved in teichoic acid biosynthesis, conferred resistance to phage 9183 but increased susceptibility to ampicillin, ceftriaxone, and daptomycin [44].

These findings suggest that the selective pressure exerted by phages can alter bacterial resistance profiles—the so called ‘phage steering’—opening new therapeutic avenues [84]. Such insights are of great importance, as they provide a potential pathway to exploit phage-induced vulnerabilities in bacteria, making them more susceptible to existing antibiotics. The use of phage cocktails and their combination with antibiotics represents a promising frontier in the treatment of multidrug-resistant bacterial infections. By broadening the host range, combating resistance, and enhancing bactericidal activity through synergistic effects, these approaches offer new hope for tackling *E. faecium* infections that are otherwise difficult to treat. However, as the research shows, the success of these strategies hinges on careful selection of phages, understanding strain-specific interactions, and optimizing combinations with antibiotics. Future research into the molecular mechanisms of synergy, alongside clinical studies, will be critical in realizing the full potential of phage–antibiotic therapies in combating antibiotic-resistant pathogens. A comprehensive summary of in vitro assays evaluating the efficacy of bacteriophages against *E. faecium* is provided in Table 1.

## 4. Ex Vivo Assessment of Phage Activity Against *E. faecium*

### 4.1. Overview of Ex Vivo Studies

Ex vivo studies occupy a critical space in phage therapy research, providing insights that bridge in vitro findings and in vivo applications. By employing biological environments that closely resemble physiological conditions, these models help to evaluate phage efficacy, biodistribution, and other therapeutic parameters with an approach that better mimics in vivo conditions while utilizing simpler experimental models. However, the limited number of these studies underscores a crucial gap.

An epithelial cell colonization model using mouse-derived 3T3 epithelial cells studied by Melo et al. allowed the study of phage efficacy against bacterial colonization on host tissues. Phages vB_EfaS-Zip (Zip), which targets *E. faecium* and vB_EfaP-Max (Max), that targets *E. faecalis*, were tested in this model to assess their ability to reduce Enterococci colonization and protect host cell viability. They also studied a biofilm model using bovine tendon collagen to emulate the wound bed environment (CWM), allowing researchers to examine phage activity within a biofilm structure like those found in chronic wounds. Phages Zip and Max were evaluated both individually and combined. This model provided a controlled environment to assess how well these phages reduce bacterial load in biofilms under wound-like conditions [48].

Another example of an ex vivo model is the simulated endocardial vegetation (SEV) system studied by Kunz Coyne et al., which replicates the infected blood clot formation typical of endocarditis. This model allows phages to be assessed under high bacterial inoculum conditions similar to those seen in infectious endocarditis, where *E. faecium* is particularly problematic. Phages NV-497 and NV-503-01, both targeting *E. faecium*, were used individually and as a cocktail in combination with antibiotics like daptomycin and ceftaroline, supporting the evaluation of phage–antibiotic synergies [85].

Ex vivo models serve as a valuable platform for investigating phage therapy in more physiologically relevant conditions, offering insights into phage effectiveness and biodistribution that are crucial for advancing clinical phage applications. We now individually assess each ex vivo model presented in this section. A summarized analysis containing key results about these models is provided in Table 2.

### 4.2. Epithelial Cell Colonization Model (3T3)

This analysis evaluates the effectiveness of bacteriophages Max and Zip against Enterococci faecalis Efa1 and *E. faecium* C410 using the 3T3 epithelial cell model, offering a controlled environment to explore phage-bacterial dynamics and viability in mammalian cells [48].

In the study setup developed by Melo et al. [48], 3T3 cells were infected with either Efa1 or C410 at a bacterial concentration of 10^8^ CFU/mL. Two hours post-infection, the cells were treated with 10^7^ PFU/mL of the respective phage—Max targeting *E. faecalis* and Zip targeting *E. faecium*. Bacterial load and 3T3 cell viability were assessed at 6 and 24 h post-treatment. Results revealed a substantial reduction in bacterial concentration in the infected 3T3 cells six hours following phage treatment. Both Max and Zip achieved approximately 3 log CFU/mL reductions in bacterial counts, which suggests a marked lytic effect of phages in the 3T3 cell model. This outcome indicates a high efficacy of phage therapy in reducing bacterial colonization in this cellular environment.

An increase in viable 3T3 cell numbers was observed six hours after treatment with phage Zip, in contrast to untreated controls. At the 24 h mark, only epithelial cells treated with either Max or Zip remained viable, signifying a lasting, beneficial effect of phage treatment on cell viability. This result suggests not only the effectiveness of the phages in bacterial elimination but also a sustained impact on cellular integrity and survival over time. However, the study does not provide detailed data on the stability or bioavailability of phages in the 3T3 model and authors note the absence of specific assessments related to environmental influences such as pH fluctuations, enzymatic degradation, or other biological barriers that may affect phage stability like immune components.

On the other hand, safety assessments were considered. Through cytotoxicity assays, Max and Zip phages were determined to not be inherently toxic to 3T3 cells. Notably, toxicity was observed when phage Zip lysate was used at concentrations of 10^8^ PFU/mL. However, purification via polyethylene glycol (PEG) eliminated this toxicity, suggesting it may have stemmed from bacterial components in the lysate rather than from the phage itself.

While the 3T3 epithelial cell colonization model provides preliminary insights into phage dosing and effectiveness, it falls short in predicting clinical outcomes due to the absence of immune interactions. Nevertheless, the insights gained here contribute valuable data for designing in vivo studies that may better elucidate the full therapeutic potential of phages in treating bacterial infections.

### 4.3. Collagen Wound Model (CWM)

The following analysis provides a detailed examination of the efficacy of phage treatment against biofilms, leveraging the collagen wound model (CWM) to evaluate phage performance under conditions simulating infected wounds [48]. An array of experiments performed by Melo et al. [48] explored the lytic effectiveness of phages Max and Zip.

An essential component of evaluating phage persistence involves assessing their stability under various environmental conditions in the CWM model. Phages Max and Zip were evaluated across a range of temperatures and pH levels, reflecting the thermal and chemical variability typical of wound sites. Both phages remained stable from −20 °C to 37 °C; however, their stability was compromised at higher temperatures, with significant titer reductions at 50 °C and complete inactivation of Zip at 60 °C.

In terms of pH stability, both phages tolerated environments from pH 5.0 to 11.0, retaining activity within this range. Nevertheless, exposure to extreme pH values (1.0, 2.0, and 13.0) resulted in complete phage inactivation. These stability findings are particularly relevant for wound settings, as wound pH can range between 4.7 and 9.0, and temperature may rise locally in response to infection. Therefore, the ability of Max and Zip to maintain stability in these ranges suggests that they could retain their lytic activity under typical wound conditions, supporting the potential persistence of phage activity within the CWM.

In regards of the lytic activity, a first experiment was conducted in parallel to evaluate the lytic activity of each phage independently, testing Max and Zip with biofilms formed on the CWM model with *E. faecalis* and *E. faecium*, respectively. Max displayed its effectiveness against *E. faecalis*. Particularly, Zip achieved a bacterial reduction of approximately 1.5 log CFU/mL between 3 and 6 h post-infection, slightly increasing to 2 log CFU/mL by 8 h. However, at 24 h, the bacterial load returned to levels similar to those in the control biofilms. Zip’s efficacy declined over time, suggesting adaptive resistance development within the biofilm structure.

A second experiment assessed a combined phage cocktail of Max and Zip against multi-species biofilms containing both *E. faecalis* and *E. faecium* within the CWM. This cocktail approach initially produced a robust reduction in bacterial load, reaching approximately 2.5 log CFU/mL after 3 h of infection. While this reduction was sustained at 6 and 8 h, the lytic efficacy gradually declined, with the 24 h sample indicating only a 1 log CFU/mL decrease in bacterial load. This mixed-phage approach showed greater initial effectiveness than single-phage applications, especially in the context of multi-species biofilms, yet it similarly faced a decrease in efficacy over time, highlighting an ongoing challenge in preventing the development of biofilm resistance to phage treatment.

The CWM ex vivo model offers valuable insights into the potential of phage therapy by enabling the estimation of bacterial load reduction and phage stability under wound-like temperature and pH conditions, informing phage formulation and dosing strategies for wound infections. Phages Max and Zip exhibit promising effects against Enterococci biofilms in this model, achieving significant bacterial reduction and stability within conditions typical of wound sites. However, limitations such as the absence of immune components, biodistribution factors, and wound-specific features like hypoxia and exudate emphasize the need for in vivo studies to validate and build upon CWM findings for clinical application in biofilm-associated infections.

### 4.4. Simulated Endocardial Vegetation (SEV) Model

In this section, we will analyze the simulated endocardial vegetation (SEV) model that used the clinical isolate R497 (DAP-R) and examined the efficacy of combined antibiotic and phage treatments, bacterial load reduction, phage lytic capacity, and resistance prevention [85].

In this study, SEV-infected clots with *E. faecium* R497 received humanized doses of daptomycin (DAP, 10 mg/kg every 24 h) and ceftaroline (CPT, 600 mg every 8 h), either alone or combined with phages NV-497 and NV-503-01 (administered individually or as a cocktail). Each phage dose was injected every 24 h at a multiplicity of infection (MOI) of 1, and bacterial load was evaluated over a 96 h period in terms of colony-forming units per gram (CFU/g). Results revealed that in treatments using DAP-CPT with individual phages (NV-497 or NV-503-01), initial bacterial killing was evident, reducing bacterial counts to 4.22 and 4.50 log10 CFU/g at 32 h, respectively. However, these regimens showed bacterial regrowth between 48 and 96 h, reaching 7.40 and 7 log10 CFU/g, respectively, by the end of the study.

Furthermore, the DAP-CPT combination with the phage cocktail NV-497-NV-503-01 produced the highest bactericidal and synergistic effect, reducing bacterial viability from an initial load of 8.77 log10 CFU/g to 3 log10 CFU/g—a 5.77 log10 CFU/g reduction. This combination achieved the greatest bacterial load reduction compared to the next most effective regimen, DAP-CPT with a single phage, NV-503-01. Notably, the DAP-CPT-phage cocktail combination induced re-sensitization of the R497 isolate to DAP, decreasing the minimum inhibitory concentration (MIC) from 16 mg/mL to 4 mg/mL after 48 to 96 h of exposure. Resistance to phages NV-497 and NV-503-01 was observed in SEV model samples after 96 h of treatment with either DAP or CPT monotherapy combined with the phage cocktail, and with the phage cocktail alone. However, the DAP-CPT-phage cocktail combination effectively prevented the emergence of phage resistance, suggesting that combined regimens may mitigate resistance development, enhancing therapeutic potential.

The SEV model does not incorporate immune components, which are essential for replicating the complex in vivo interactions of endocardial vegetations. Furthermore, it did not specifically address phage persistence or degradation mechanisms and factors such as pH or enzymatic activity. The study did not address phage safety either. In conclusion, the SEV model provides a foundational understanding of phage therapy in a controlled environment and illustrates the potential for phage–antibiotic synergy in treating resistant infections. However, to fully harness phage therapy’s clinical potential, further studies in complex models that incorporate the immune system are essential.

## 5. Efficacy of In Vivo Studies on Phage Therapy for *E. faecium*

### 5.1. Overview on Key Insights

In vivo studies are fundamental in advancing phage therapy by providing insights into the efficacy, safety, and bioavailability of bacteriophages within living organisms. Unlike in vitro studies, in vivo experiments facilitate the observation of complex interactions between phages, host bacteria, and the immune system, allowing for a more realistic understanding of phage dynamics in biological environments. These studies are therefore indispensable for comprehensively assessing phage therapy.

To date, only the application of two animal models about phage therapy efficacy against *E. faecium* have been reported. On one hand, the use of *Galleria mellonella* larvae has gained traction in preliminary studies for phage therapy due to its affordability, ease of handling, and ease of approval as it does not require an ethics committee review. This model offers a simplified system to evaluate microbial virulence, phage efficacy, and basic safety, as well as allowing researchers to observe survival outcomes, bacterial load reduction, and other relevant parameters.

On the other hand, mouse models enable the assessment of phage efficacy and provide critical insights into optimal dosing, administration routes, and the timing of treatment initiation.

Although in vivo studies offer significant advantages for advancing phage therapy, they also present inherent limitations. Physiological and immunological differences between animals and humans complicate the direct extrapolation of results to human clinical scenarios. Variations in immune response and metabolic pathways between species can influence the efficacy and safety of phages. Additionally, the inherent complexity of in vivo studies entails higher costs and the need for rigorous experimental controls to obtain reliable results. Nonetheless, in vivo research remains essential for progressing phage therapy as a viable alternative to antibiotic treatment, particularly against multidrug-resistant *E. faecium*. The information obtained from these studies is crucial for understanding the therapeutic potential of phages and for guiding the design of future clinical trials in humans.

An overview of in vivo phage therapy studies is presented in Table 3, outlining models used, treatment effects, and important study constraints.

### 5.2. Phage Therapy Against VRE in Galleria mellonella

In an assessment of the effectiveness of phage therapy against VRE, for the first time with this in vivo model, the study by El Haddad et al. utilized *G. mellonella* larvae as a model organism for evaluating an individual phage (MDA1) and a cocktail of four phages (MDA1, MDA2, MDA3, and MDA4) to measure their efficacy against a VRE strain isolated from the feces of a hematopoietic stem cell transplant patient [86].

For experimental infection, *G. mellonella* larvae were injected with VRE at a concentration of 10^7^ CFU/10 μL, followed by administration of the phage therapy (either MDA1 alone or the phage cocktail) at a concentration of 10^6^ CFU/10 μL.

The study divided the larvae into two principal groups: a prevention group (PG), which received phages one hour prior to VRE infection, and a treatment group (TG), which received phages one hour post-infection. Control groups included larvae injected only with bacteria, those injected with only phages, larvae injected with sterile medium to assess the effects of injection trauma, and untreated larvae. Each group was composed of five larvae, with health assessments conducted eight hours post-incubation at 37 °C using a standardized health index scoring system [87].

Key results showed a significant increase in survival rates among larvae treated with phage therapy. In contrast, only 32% of the VRE-infected larvae survived without phage intervention, and survival rates exceeded 80% in both the prevention and treatment groups following phage administration. Notably, the phage cocktail proved more effective than the individual MDA1 phage, achieving a survival rate of 82% compared to 66% with MDA1 alone. Furthermore, the administration of phages alone demonstrated no toxicity, as survival rates among larvae receiving only phages were equivalent to those observed in larvae injected with sterile medium or left untreated, underscoring the safety of the phage therapy. The study did not report any adverse outcomes associated with phage treatment. While the study yielded substantial evidence of phage efficacy against VRE in improving survival rates with VRE infections and exhibiting the safety of the administered phages, it did not explore the persistence or biodistribution of phages, limiting insights into the pharmacokinetics of phage therapy in this invertebrate model. The study also did not include findings on phage interactions with the host immune system, such as potential immune activations or inhibitions following phage administration.

### 5.3. Phage Cocktail Efficacy in Galleria mellonella

Another study by El Haddad et al. focused on detailing the efficacy of two specific bacteriophages against VRE in *G. mellonella* larvae. Two phages, MDA1 and MDA2, were both confirmed free of virulence genes, antibiotic resistance genes, and integrase genes, supporting their therapeutic safety. These phages were combined into a cocktail to enhance effectiveness against VRE infection in the larvae model [53]. For experimental infection, larvae were injected with VRE strain VRE004 at a concentration of 10^7^ CFU/10 μL. In the treatment group, the phage cocktail was administered one hour post-bacterial infection, at a concentration of 2 × 10^6^ PFU/10 μL.

In the prophylactic group, the cocktail was given one hour before VRE infection. Control groups included larvae injected with only bacteria, only phages, sterile medium, and untreated larvae. Each group comprised eight larvae, with five replicates to ensure consistency. Larvae health and mortality were monitored over a 48 h incubation at 37 °C.

This study indicated that the phage cocktail was non-toxic to the larvae. The survival rate of larvae injected with only the phage cocktail (85%) was similar to that of larvae injected with saline (82.5%), confirming that the phage administration alone did not reduce survival. In terms of therapeutic efficacy, the larvae in both the treatment and prophylactic groups had a significantly higher survival probability compared to larvae exposed to VRE alone. Specifically, the treatment group exhibited a 3.7-fold increased survival probability (OR = 3.7, SE = 1.8, *p* = 0.07), while the prophylactic group’s survival probability was 6.5 times higher (OR = 6.5, SE = 3.3, *p* < 0.001). Furthermore, a 16S analysis revealed a significantly reduced bacterial load of VRE in the phage-treated groups relative to the VRE-only group, highlighting the bactericidal efficacy of the phage cocktail.

No adverse outcomes associated with phage therapy were noted, underscoring its potential as a safe treatment option against VRE infection in this model. The study did not provide specific insights into the persistence or biodistribution of the phage cocktail within the *G. mellonella* model. This organism possesses an innate immune system that shares similarities with vertebrate immune systems, making it a viable model for assessing microbial virulence and the efficacy of antimicrobial agents [88,89]. However, the study did not examine potential interactions between the phages and the host immune system. Information on immune responses, including potential activation or inhibition by the phages, could shed light on the broader mechanisms by which phage therapy exerts its effects and may impact the phages’ antibacterial efficacy.

In conclusion, the *G. mellonella* model presents a useful alternative for initial phage therapy evaluation, with this study providing evidence for safety and efficacy in managing VRE infections. Nonetheless, further studies are essential to confirm these findings in more advanced preclinical and clinical models to develop phage therapy as a viable alternative for managing multidrug-resistant infections.

### 5.4. Survival Benefits of Phage vB_EfaH_163 in Galleria mellonella

The efficacy of phage vB_EfaH_163 in controlling the growth of VRE was assessed by Pradal et al. in vitro and in vivo using the *G. mellonella* model. In vitro results are presented and summarized in Section 3.1 and Table 1 [73].

In vivo, the lethality of *E. faecium* VR-13 was initially assessed by injecting *G. mellonella* larvae with bacterial concentrations of 10^5^, 10^6^, or 10^7^ CFU per larva. Each injection was administered in 10 μL volumes to the larvae’s right proleg, while a control group was injected with PBS to evaluate baseline survival. Larval mortality was monitored over five days, establishing a lethal dose to model bacterial infection. To assess the therapeutic potential of phage vB_EfaH_163, larvae were inoculated with *E. faecium* VR-13 at a concentration of 10^5^ CFU/larva. One hour post-infection, a phage suspension—prepared by PEG-NaCl concentration and resuspended in sterile distilled water—was injected into the opposite proleg at an MOI of 0.1. A control group received only distilled water, and larval survival was monitored for five days.

*E. faecium* VR-13 infection led to substantial larval mortality, with all larvae injected with 10^7^ CFU/larva succumbing within 48 h. Larvae challenged with 10^5^ and 10^6^ CFU/larva showed mortality rates of 20% and 30%, respectively, after five days. Overall, infection with VR-13 resulted in approximately 40% larval mortality by the end of the five-day period. Treatment with phage vB_EfaH_163 at MOI 0.1 increased larval survival by 20%, though this increase was not statistically significant, suggesting a limited therapeutic effect under these conditions.

These findings highlight both the potential and the limitations of vB_EfaH_163 in controlling VRE. While the phage demonstrated notable antibacterial activity in vitro, this effect was transient, with the bacterium resuming growth within 24 h. Similarly, phage treatment increased survival in infected larvae, albeit without statistically significant results. These limitations underscore the need for further research into optimizing phage application parameters to enhance therapeutic outcomes.

The study acknowledges that *Galleria mellonella* serves as an effective model for assessing VRE virulence and testing antimicrobial treatments due to its innate immune system, which shares structural and functional similarities with vertebrate immune responses. However, no specific details were provided on how vB_EfaH_163 interacts with the immune system of *G. mellonella* during infection. Observations of immune activation or suppression, or interactions with other biological components, were not documented in this study. Understanding immune–phage interactions in this model could provide insight into the mechanisms by which phage therapy exerts its antibacterial effects and influence host survival.

The data overall supports the use of *G. mellonella* as a non-mammalian, ethical, and cost-effective model to preliminarily assess phage therapy efficacy. However, additional studies are necessary to optimize phage selection, dosing, and delivery methods for effective and sustained bacterial control.

### 5.5. BALB/c Mouse Model for E. faecium Bacteremia

The study by Biswas et al. in 2002 investigated the efficacy of phage therapy in a murine model of vancomycin-resistant *E. faecium* bacteremia for the first time, focusing on the phage ENB6 as a potential therapeutic intervention to rescue infected mice from a lethal VRE challenge [37].

The study utilized one-month-old female BALB/c mice and aimed to establish whether phage treatment could provide effective protection against bacteremia induced by a lethal dose of VRE. To induce bacteremia, the mice were injected intraperitoneally (i.p.) with a clinical strain of VRE, CRMEN 44, at a concentration of 10^9^ CFU, a dose determined in preliminary studies to be the minimum lethal dose (MLD). Following bacterial challenge, ENB6 was administered in a single i.p. injection across a range of concentrations and at various post-infection time-points to assess dose dependency and the impact of treatment timing on survival outcomes. The study design included two main experiments: a dose range study and a delayed treatment study. In the dose range assessment, ENB6 was injected 45 min post-infection at five concentrations: 3 × 10^9^, 3 × 10^8^, 3 × 10^6^, 3 × 10^4^, and 0 PFU. The delayed treatment study evaluated the highest dose of ENB6 (3 × 10^9^ PFU) at intervals of 5, 8, 12, 16, and 24 h after infection to determine the effect of treatment timing on therapeutic success.

Key results indicated that a single high-dose injection of ENB6 at either 3 × 10^9^ or 3 × 10^8^ PFU rescued all mice in the VRE bacteremia model, even when treatment was delayed by up to five hours post-infection. Lower doses or more extended delays in phage administration however correlated with reduced survival rates. Remarkably, even with treatment delayed up to 24 h, approximately half of the mice—though in a moribund state—were successfully rescued by a single phage injection. In addition to enhanced survival, phage therapy led to a 200-fold reduction in blood bacterial counts within 20 h of infection compared to control animals treated with PBS. Treated mice also demonstrated an improvement in clinical health scores relative to the untreated controls, providing further evidence of the therapeutic benefit of ENB6.

Certain limitations were also observed. Survival rates declined in mice treated with lower phage doses or when treatment was administered after extended delays, suggesting that both dose and timing are critical factors in optimizing phage therapy efficacy. Additionally, the study noted that stressed animals could exhibit increased sensitivity to phage ENB6 or residual endotoxins within phage preparations, which might impact treatment outcomes under certain conditions.

In a complementary experiment, the study compared the effectiveness of phage C33 with ENB6 against a strain of *E. faecalis* (CRMEN 19) that was resistant to ENB6 but susceptible to C33. While C33 treatment led to 100% survival, ENB6 treatment was only 20% effective—an outcome even lower than that in the control group. This result highlighted the strain specificity of phage efficacy, with ENB6′s limited impact attributed to its lack of lytic activity against the *E. faecalis* strain CRMEN 19.

On the topic of phage persistence and biodistribution, the source provided limited insight. It references a 1973 pharmacokinetic study indicating that phages are rapidly cleared from the bloodstream, subsequently accumulating in the spleen and other reticuloendothelial organs, where they may remain viable for at least a week [90]. However, this study did not investigate the persistence of ENB6 and C33 specifically or document their distribution across mouse tissues, leaving these aspects open for future exploration.

The study also briefly examined the interaction of ENB6 with the host immune system. ENB6 was found to elicit a humoral immune response, with significant increases in IgG and IgM levels following repeated injections in mice. Importantly, this immune response did not lead to anaphylaxis or other adverse events, suggesting that ENB6 could be safely administered without triggering severe immune-related side effects. However, the potential impact of this immune response on long-term phage therapy efficacy was not directly assessed. The study did propose that selecting and presenting phages in ways that minimize immune recognition could enhance treatment outcomes, particularly for chronic infections where repeated dosing might otherwise induce immune resistance to the phage.

Though no specific adverse outcomes from the phage-immune interactions were noted, the study underscores the need for further research to fully understand how immune responses might influence the therapeutic efficacy of phages. Insights from such studies could support the development of phage variants less prone to immune recognition, thereby optimizing their utility in clinical settings.

### 5.6. Hydrogel-Encapsulated Phage Therapy in BALB/c Mice

The use of mouse models has provided insights into the in vivo efficacy, persistence, biodistribution, and immune interactions of phage therapy. In a recent study by Khazani Asforooshani et al., the therapeutic potential of phage EF-M80 encapsulated in hydrogel to treat wound infections caused by *E. faecium* in BALB/c female mice was assessed [57].

These animals were divided into six experimental groups to evaluate various aspects of phage treatment: the healthy control group (HC), consisting of uninjured, untreated mice; a negative control group (NC), which received sterile distilled water on wounded skin; a positive control group (B), where mice were wounded, infected, and left untreated; a phage-treated group (Ph), receiving phage EF-M80 on infected wounds; a hydrogel-encapsulated phage group (Ph-hyd), where infected wounds were treated with hydrogel containing phages; and a hydrogel-only control group (Hyd), with infected wounds treated with hydrogel lacking phages.

The infection model involved creating a full thickness wound on the mice’s dorsal skin, with a 5 mm biopsy punch, followed by inoculation of the wound area with an *E. faecium* suspension. After a 24 h period, phage EF-M80 was administered at an optimal multiplicity of infection (MOI) of 0.1. The Ph-hyd group received the phage in hydrogel form, the Hyd group received hydrogel without phages, the NC group was treated with sterile distilled water, and the B group remained untreated. Wound healing was then monitored over a 14 day period.

Results from this study demonstrated significant differences in wound healing rates across treatment groups. The Ph-hyd group achieved complete wound closure by day 14, with notable healing observed as early as day 3, suggesting that phage therapy in a hydrogel delivery system substantially accelerates wound recovery. The Ph group also showed faster healing compared to the NC and B groups, with substantial wound closure observed by day 14, though at a slower rate than Ph-hyd. These findings indicate that while phage therapy alone aids wound repair, its efficacy is enhanced with a controlled-release delivery system, like hydrogel. In contrast, the NC group exhibited delayed wound closure compared to Ph and Ph-hyd, with a sizable wound area persisting by day 10, while the slowest healing was observed in the untreated B group, emphasizing the negative impact of untreated infection on wound repair.

Histopathological analysis further supported these findings. Tissue samples collected on day 14 revealed that the B and NC groups displayed incomplete epidermal regeneration, with significantly increased epidermal thickness compared to the Ph-hyd group. Both HC and Ph-hyd groups maintained normal epidermal thickness, indicating efficient tissue repair. Additionally, essential skin structures such as hair follicles and blood vessels in hydrogel-treated groups (Ph-hyd and Hyd) showed morphology closely resembling that of healthy skin, suggesting that hydrogel aids in the restoration of these structures, potentially contributing to the faster re-epithelialization and wound healing observed in these groups. Higher fibroblast density, a marker of skin regeneration and proliferation, was observed in both Ph-hyd and Ph groups relative to control groups, highlighting the beneficial role of phage therapy in tissue regeneration. Inflammatory markers, such as neutrophil presence, were significantly lower in the Ph-hyd group compared to the positive control, suggesting that the hydrogel delivery system was effective in reducing inflammation at the wound site. No other specific negative outcomes regarding phage therapy efficacy were reported in this mouse model.

Regarding interactions with host immune factors, this study does not describe how phages interact with the mouse immune system, nor does it provide examples of immune responses in mice or how these might influence phage efficacy. Further investigation into immune interactions could illuminate potential barriers or facilitators in the therapeutic use of phages, especially in cases where immune responses might alter phage effectiveness over prolonged treatments.

## 6. Phage Therapy Against *E. faecium* in Patients

### 6.1. Overview of Human Studies

As previously stated, *E. faecium* is one of the ESKAPE pathogens classified as high priority on the WHO list of priority pathogens. This bacterium is part of the human gastrointestinal microbiota but can cause invasive infections, especially in immunocompromised hosts. *E. faecium* is usually resistant to ampicillin and its treatment of choice is usually vancomycin.

Colonization and subsequent infections by multidrug-resistant bacteria in patients are associated with high mortality, and therapeutic strategies with antibiotics sometimes have low success rates. The treatment of vancomycin-resistant enterococci is not well defined and is a challenge. In these cases of multidrug-resistance, phage therapy has become a possible therapeutic option, especially in infections that do not respond to antibiotic treatment. Unfortunately, to date, no phage therapy products have been commercialized in the market, and these are phages that are administered as compassionate use because they are not yet approved for daily use.

There are only three recent published cases that have demonstrated in vivo efficacy of phage therapy for *E. faecium* infections in humans that do not respond to conventional antibiotic therapy [91,92,93]. In these studies, no serious, mild, or unexpected side effects of phage therapy were observed in the treated patients. These clinical cases are summarized in Table 4.

### 6.2. Bacteriophage Rescue in Pediatric VRE Infection Post-Transplantation

Kevin Paul et al. reported the first case of intravenous phage therapy against *E. faecium* infection [91]. This case was reported also by Jean-Paul et al., which integrates the outcomes of personalized bacteriophage therapy in 100 cases [94]. The case presented by Kevin Paul et al. shows a pediatric patient with a severe clinical picture, with cirrhosis, multiple liver abscesses, and recurrent cholangitis, who was evaluated for liver transplantation because of continued deterioration. After transplantation, an extensive intrahepatic bacterial colonization resulted in severe systemic infection, caused mainly by vancomycin-resistant *E. faecium*. However, despite repeated abdominal washings and revision of the biliodigestive anastomosis, the *E. faecium*-related abdominal site infection did not resolve, and the hepatic ischemia injury resulted in superinfected necrotic areas. Due to the progressive damage to the organ, and to eradicate the reservoir of infection, two more transplants had to be carried out subsequently weeks later. However, *E. faecium* was repeatedly detected, and the patient’s health status deteriorated.

Since multiple antibiotic treatment regimens and extensive surgical treatment were not sufficient to control the infection, a personalized cocktail of two phages was used: EFgrKN and EFgrNG. Two daily injections of these phages were administered. Ten days of 2 mL/kg bodyweight (BW) as a magistral preparation (joint titer first batch 8.1 × 10^7^ PFU/mL in NaCl 0.9%) followed directly by another ten days of 2 mL/kg BW (joint titer second batch 5.2 × 10^8^ PFU/mL in NaCl 0.9%). The phages were administered intravenously for 20 days. It should be noted that there was a synergistic effect of phage EFgrKN in combination with vancomycin. Even shortly after the application of the phage, *E. faecium* was identified with a vancomycin-susceptible phenotype. During one year of follow-up, treatment with phage therapy was not associated with any adverse events and the patient improved clinically.

### 6.3. Phage–Antibiotic Synergy in Recurrent VRE Bacteremia Management

In the study of Madison et al., systemic antibiotics alone were unable to achieve durable remission of recurrent *E. faecium* bloodstream infections in a 57-year-old female [92].

This patient became unresponsive to antibiotic combination therapy over the course of 7 years. A personalized cocktail of two phages, siphovirus phages Φ9184 and ΦHi3, was added intravenously and orally to the patient’s antimicrobial regimen. On day 182, three times Φ9184 daily at 1 × 10^9^ PFU per dose and on day 303, ΦHi3 was added to the treatment regimen alongside Φ9184 for a total of 2 × 10^9^ PFU/dose.

After phage therapy, the patient improved and the intestinal burden of E. faecium was reduced. The patient experienced fewer number of hospitalizations and a better quality of life for six months. However, the patient passed away due to pneumonia 7.5 months after discontinuation of phage therapy. This study demonstrates the potential of phages as an additional antimicrobial option for the treatment of invasive enterococcal infections and underscores the need for further research.

### 6.4. Phage Therapy in Cardiothoracic Surgery-Associated Infections

In the study of Evgenii et al., they collected several cases of phage therapy including one case for *E. faecium* [93]. In this case, the patient was in critical condition due to an infected aortic arch prosthesis two years after a previous aortic dissection. In the following two years, the patient was admitted for repeated graft infection. As a consequence, the patient developed pleural empyema followed by purulent infection of a bronchial tree through a fistula caused by *E. faecium* and two other bacterial isolates (*S. aureus* and *P. aeruginosa*). The infection did not respond to conventional antibiotic treatment. A phage cocktail was administered for two days (1 × 10^8^ PFU/mL of *Staphylococcus* phage CH1, *Enterococcus* phage Enf1, and *Pseudomonas* phages, PA5 and PA10), once through a pigtail drainage and, on the same day, once orally. Two days later, the patient underwent thoracotomy and decortication of the pleural empyema, and a phage cocktail was locally applied intraoperatively.

The patient showed elevated CRP levels shortly after phage treatment, which decreased in the following days, as was reported previously in other patients with phage therapy. This could be explained either by normal postoperative conditions or by significant bacterial lysis due to phage therapy. After the second phage application, *S. aureus*, *E. faecium*, nor *P. aeruginosa* were detected and phage therapy was stopped. Unfortunately, the patient passed away two months after phage therapy due to a new infection by *E. coli* and *P. aeruginosa*.

## 7. Discussion

The increasing amount of research on phage therapy in recent years highlights the growing need for alternative treatments beyond traditional antibiotics, especially given the significant MDR infection levels noted with *E. faecium*. However, certain challenges in the development and application of phage therapy remain unclear, which could impact its implementation in clinical practice.

One of the principal advantages of phage therapy is the specificity of phages, offering an alternative that targets pathogen strains while preserving commensal microbiota, minimizing the risk of dysbiosis in the host. However, this specificity also presents a major limitation as the narrow host range of individual phages entails the development of phage cocktails tailored to each bacterial strain. Phage specificity precludes phage therapy from being broadly applied without tailored interventions. This process of creating and optimizing phage cocktails often requires time-intensive isolation and characterization steps, which can delay treatment in acute infections. The establishment of pre-characterized phage libraries, potentially containing phage genomic sequences—discarding the presence of resistance, lysogeny, or virulence genes—and infective in vitro characterization, could help address this issue by shortening the time between diagnosis and treatment [95]. However, there is limited data on the efficacy of such libraries and further studies are needed to optimize their design and implementation, from isolation to transport and administration.

An additional challenge in phage therapy is the potential for bacteria to develop resistance to phages, similar to the resistance observed with antibiotics. As reviewed, phage cocktails could reduce the emergence of resistant colonies, or, in a more efficacious approach, a combination of phages plus antibiotics. Studies suggest that phage–antibiotic combination therapy may offer advantages over single or cocktail phage treatments. For instance, while phage cocktails are designed to broaden the range of target strains and mitigate the emergence of phage resistance, phage–antibiotic combinations introduce additional mechanisms of action. This dual approach can induce synergistic effects, including bacterial re-sensitization to previously ineffective antibiotics, as seen in several studies where phages disrupt biofilms or cellular defenses, enhancing antibiotic efficacy [96].

Importantly, this strategy can reduce the therapeutic reliance on large phage libraries, potentially simplifying treatment logistics. Additionally, certain antibiotics have been shown to facilitate phage entry or replication by altering bacterial cell physiology, while phages can expose bacteria to selective pressure, weakening their defenses against antibiotics. This has been demonstrated in cases involving *E. faecium*, where phage–antibiotic synergy achieved substantial reductions in bacterial load and improved therapeutic outcomes [25].

However, caution is warranted, as not all phage–antibiotic combinations yield synergistic results. As reviewed, some phage–antibiotic combinations may exhibit antagonistic interactions, depending on the mechanisms of action and bacterial strain involved. Therefore, while combination therapy offers significant potential, further studies are essential to optimize these strategies and elucidate the molecular underpinnings of their interactions. Comparative studies of combination therapy versus phage cocktails would provide valuable insights into their respective efficacies, costs, and impacts on resistance dynamics and microbiota preservation.

The findings from in vivo studies have shown promising results regarding the effectiveness, pharmacokinetics, and safety of phage therapy against *E. faecium*. In general, phage therapy improved survival rates and demonstrated significant reductions in bacterial load. Although in vivo models provide valuable insights, differences between animal and human immune responses present a key limitation, as these may impact the persistence and biodistribution of phages in human patients. As some authors have discussed, current knowledge gaps exist on the appropriate routes of administration, phage selection, frequency of administration and dosage, interactions with other phages, emergence of phage resistance, inadequate phage delivery, and superhost immune response are some of the factors that could potentially result in phage therapy failure [97,98].

Another important factor to consider is the regulatory environment surrounding phage therapy, which varies by region and may restrict the speed of phage development and clinical trials. While phages can be used as compassionate treatment in cases where conventional antibiotics have failed, increasing clinical trials will be essential to establish phage therapy as a viable alternative in routine clinical practice. Robust clinical data are needed to validate and standardize phage treatments that could lead to the development of products that meet pharmaceutical-grade manufacturing standards.

To conclude, phage therapy against *E. faecium* offers considerable potential, particularly as antibiotic resistance continues to rise. However, challenges such as the need for phage cocktails, the development of bacterial resistance to phages, and the complexity of scaling phage therapy for clinical use must be addressed to enable its wider application. Future research efforts should focus on developing phage libraries, optimizing phage–antibiotic combinations, and conducting comprehensive clinical trials. With continued advancement in these areas, phage therapy could become a crucial component in the treatment of MDR infections, especially in cases where conventional therapies have proven insufficient.

## Figures and Tables

**Table 1:** Summary of in vitro studies evaluating phage therapy against *E. faecium*.

No.	Phage	Bacteria Strain	Assay Type	Outcome	Synergistic Effects (Cocktails/Antibiotics)	Conclusion	Ref.
1	IME-EFm5	4P-SA (VRE clinical strain)	Plate assay	Clear plaque formation	Not evaluated.	Lytic phage specific to 4P-SA. Narrow host range. Purified endolysin LysEFm5 is promising.	[40]
2	113	ATCC 19950-B1, R497 (VRE, DAP-R), HOU503 (VRE, DAP-SDD)	Modified plate assay, TKA, phage resistance assay	Synergy and bactericidal activity with phage-DAP-AMP, phage-DAP-CPT, and phage-DAP-ERT combinations.	Phage + β-lactam combinations effective against biofilms and resistance prevention.	Effective against biofilm infections, prevents resistance.	[74]
3	113	R497 (VRE, DAP-R), VRE, DAP-SDD HOU503, others	Modified checkerboard, TKA, biofilm eradication	Medium susceptibility in 6/8 strains; high in R497, low in HOU503.	DAP + phage cocktail showed synergy. Prevented resistance in HOU503.	DAP + AMP with phage cocktails eradicated MDR biofilm.	[75]
4	NV-497	R497, HOU503, others	Modified checkerboard, TKA, biofilm eradication	Medium susceptibility in 4/8 strains. High in 3/8 strains and low in 5938.	Synergy with DAP + AMP in cocktails.	Phage cocktail with DAP + AMP eradicated MDR biofilm.	[75]
5	NV-503-01	R497, HOU503, others	Modified checkerboard, TKA, biofilm eradication	Medium susceptibility in 4/8 strains. High in 3/8 strains and low in strain 5938.	Synergy with DAP + AMP in cocktails.	Phage cocktail with DAP + AMP eliminated MDR biofilms.	[75]
6	NV-503-02	R497, HOU503, others	Modified checkerboard, TKA, biofilm eradication	Medium susceptibility in 4/8 strains. Low in 3/8 strains and High in SF12047.	Synergy with DAP + AMP in cocktails.	Phage cocktail with DAP + AMP eradicated MDR biofilms.	[75]
7	NV-S447-01	R497, HOU503, others	Modified checkerboard, TKA	No susceptibility in 4/8 strains. Medium in 3/8 and high in S447.	Not evaluated.	Not tested further due to low efficacy.	[75]
8	NV-S447-02	R497, HOU503, others	Modified checkerboard, TKA	No susceptibility in 4/8 strains. Medium in 3/8 and high in S447.	Not evaluated.	Not tested further due to low efficacy.	[75]
9	9181	R497, HOU503, others	Phage sensitivity assay	No susceptibility in 6/8 strains. Low in R497 and 5938.	Not evaluated.	Not tested further due to low efficacy.	[75]
10	9183	R497, HOU503, others	Phage sensitivity assay	No susceptibility in 6/8 strains. High in R497, low in 5938.	Not evaluated.	Not tested further due to low efficacy.	[75]
11	9184	R497, HOU503, others	Phage sensitivity assay	No susceptibility in 6/8 strains. High in R497 and 5938.	Not evaluated.	Not tested further due to low efficacy.	[75]
12	EfV12-phi1	TX1330	Experimental coevolution	Initial bacterial density reduction, resistance developed after 6–7 transfers.	Not evaluated.	Phage–host coevolution led to an evolutionary arms race, increasing bacterial resistance.	[51]
13	vB-EfmS-S2 (S2)	34 clinical VRE isolates	Plaque assay, biofilm inhibition	Lysed 82.3% of isolates. Stable at 37 °C, pH 7–9. Biofilm reduction.	Not evaluated.	Promising for biofilm prevention.	[43]
14	vB_GEC_EfS_9	70 clinical strains, 23 VRE strains	Spot titration, phage adsorption, pH, and temperature stability	Lysed 84% of strains. Stable at 45 °C and pH 3–12.	Not evaluated.	Broad host range with high stability, potential therapeutic candidate.	[41]
15	NV-497	R497, HOU503	Checkerboard assay, TKA	Synergy with DAP and CPT (FIC 0.5). Antagonism with LNZ and MIN.	The addition of LNZ significantly decreased the burst size of NV-497.	Phage–antibiotic antagonism observed with protein synthesis inhibitors.	[76]
16	NV-503-01	R497, HOU503	Checkerboard assay, TKA	Synergy with DAP and CPT (FIC 0.5). Antagonism with LNZ and MIN.	MIN inhibited NV-503-01 phage burst size and antibacterial activity.	Phage–antibiotic antagonism observed with protein synthesis inhibitors.	[76]
17	Athos	14 clinical VRE isolates	Plaque assay, AST	Strong lytic activity on isolation strain. Reduced teicoplanin MIC in resistant mutants.	Mutations in *epa* increase susceptibility to certain antibiotics.	Athos belongs to a new genus of virulent phages.	[54]
18	Porthos	14 clinical VRE isolates, other enterococci	Plaque assay, resistant mutant analysis	Strong lysis in related strains. Receptor remains unidentified.	Not evaluated.	Porthos is a new lytic phage with broad range and unknown receptor.	[54]
19	Aramis	14 clinical VRE isolates	Plaque assay, AST	Strong activity on two VRE strains. Reduced teicoplanin and DAP MIC in mutants.	Mutations in *epa* increase antibiotic susceptibility.	Represents a new phage genus with potential synergy in phage–antibiotic therapy.	[54]
20	D’Artagnan	14 clinical VRE isolates	Plaque assay	Lysis on two strains, including its isolation strain.	Not evaluated.	Part of a new phage genus with Aramis.	[54]
21	Planchet	14 clinical VRE isolates	Plaque assay	Strong lytic activity in two VRE strains. Host range includes three STs.	Not evaluated.	Planchet is a new species in the Denvervirus genus.	[54]
22	ATCC 19950-B1 (113)	R496, HOU503, R497	TKA	Synergy and bactericidal effects with DAP-AMP-phage and DAP-CPT-phage.	Strain-specific phage prevented resistance development.	Synergistic with antibiotics, prevented phage resistance.	[77]
23	ATCC 19950-B1 (113)	ATCC 19950, Com12, 1.141.733	TKA	Phage alone did not eradicate bacteria. DAP + phage reduced 3.0–3.5 log10 CFU/mL.	Synergy with DAP and prevention of phage 9184 resistance.	Phage 113 and DAP showed bactericidal activity and prevented resistance.	[78]
24	9184	ATCC 19950, Com12, 1.141.733	TKA	2-log10 CFU/mL reduction with phage 9184 in cocktail. Synergy with DAP.	DAP prevented phage 9184 resistance.	DAP with phage 9184 prevented resistance.	[78]
25	vB_Efm_LG62 (LG62)	16 Enterococci strains (12 *E. faecium* and 4 *E. faecalis*), including MDR, VAN-sensitive.	Plaque assay, biofilm inhibition	Lysed *E. faecium* 21112362. Inhibited biofilm formation by 30%.	Not evaluated.	LG62 has a narrow spectrum but promising biofilm inhibition.	[64]
26	iF6	*E. faecium* FS86, other strains	Plaque assay, exponential/stationary phase assays	Inhibited growth in both phases.	Not evaluated.	iF6 is a lytic phage with excellent adsorption kinetics but limited pH stability.	[55]
27	vB_Efs8_KEN04	26 MDR *E. faecalis*, 11 *E. faecium* isolates	Plaque assay, biofilm formation/disruption	Lytic against all *E. faecalis* and one *E. faecium* isolate. Biofilm disruption.	Not evaluated.	Broad range, effective biofilm disruption in *E. faecalis*.	[58]
28	STH1	*E. faecium* 1969 (VRE and TEI-R)	Plaque assay, bacterial reduction	Plaque formation, significant turbidity reduction. Latency 18 min, burst size 334 virions/cell.	Not evaluated.	Effective lytic phage with good activity and short latency.	[63]
29	Ben	*E. faecium*, *E. faecalis* (VRE and VSE)	Plaque assay, experimental coevolution	Lysis in most strains. Synergistic in cocktails.	Synergy in cocktails, suppressing growth for 72 h.	Ben shows broad range and improved efficacy in cocktails.	[79]
30	Bop	*E. faecium*, *E. faecalis* (VRE and VSE)	Plaque assay, experimental coevolution	Lysis in a wide range of strains. Synergistic in cocktails for 72 h.	Synergy in cocktails with resistance linked to *Epa* mutations.	Bop is effective in cocktails, with resistance through exopolysaccharide mutations.	[79]
31	V12	*E. faecium*, *E. faecalis* (VRE and VSE)	Drop assay, coevolution	Lysis in most strains. Synergy in cocktails for 72 h.	Synergy in cocktails.	Broad host range, effective in cocktails.	[79]
32	Bill	*E. faecium* TX1330	Coevolution, genome sequencing	Mutations in *Yqw* locus.	Not evaluated.	Induces mutations in exopolysaccharide synthesis, potential resistance mechanism.	[79]
33	Bob	*E. faecium* TX1330	Coevolution, genome sequencing	Mutations in *Yqw* locus.	Not evaluated.	May exert selective pressure leading to mutations in exopolysaccharide synthesis.	[79]
34	Carl	*E. faecium* TX1330	Coevolution, liquid culture in cocktails	Mutations in *Yqw* locus. Lower abundance in cocktails.	Synergy in cocktails.	Inhibits growth in cocktails but shows lower abundance.	[79]
35	Φ1/PlyV12	*E. faecalis*, *E. faecium*, *S. pyogenes*, *S. aureus*	OD assay, viability assay	4-log reduction in *E. faecalis* V12 in 15 min. Maximum activity at pH 6.0.	Not evaluated.	Lytic enzyme from phage Φ1 with broad activity, including vancomycin-resistant strains.	[80]
36	IME-EFm1	22 clinical isolates (2 VRE)	Host range test, growth curve	Lysed 17/22 isolates (77.3%). Latency 30 min, burst size 116 PFU/cell.	Not evaluated.	Promising, but metallo-β-lactamase gene requires further study before clinical use.	[42]
37	vB-EfmS-S2	34 clinical isolates of VRE and *E. faecalis.*	Plaque formation, biofilm inhibition	Lysed 82.3% of *E. faecium*. Stable at 30–45 °C, pH 5–11. Inhibits and disrupts biofilms.	Not evaluated.	Potential therapeutic agent for vancomycin-resistant *E. faecium* infections, including biofilms.	[43]
38	9181	10 laboratory isolates, 11 MDR clinical isolates of *E. faecium*	Phage susceptibility, synergy assay	Narrow host range. Mutations in *sagA* increased susceptibility to AMP and CTX.	Synergy with AMP and CTX.	Narrow host range, synergizes with AMP and CTX.	[44]
39	9183	10 laboratory isolates, 11 MDR clinical isolates of *E. faecium*	Phage susceptibility, synergy assay	Narrow host range. *epaR* and *epaX* mutations increase susceptibility to AMP, CTX, and DAP.	Synergy with AMP, CTX, and DAP.	Synergizes with antibiotics, especially CTX.	[44]
40	9184	10 laboratory isolates, 11 MDR clinical isolates of *E. faecium*, 1 strain of *E. faecalis*	Phage susceptibility assay	Broader host range. Resistance from capsule biosynthesis mutations.	Not evaluated.	Broader host range than 9181 and 9183. Resistance arises from capsule biosynthesis mutations.	[44]
41	vB_EfaH_163	77 *E. faecium* and 11 *E. faecalis* isolates, including clinical, animal, and environmental strains	Plaque assay, genome sequencing, stability, TKA	Broad host range. Significant reduction in *E. faecium* VR-13 during the first 7 h post-infection.	Not evaluated.	Broad host range with no genes related to pathogenicity, AMR, or lysogeny. Efficacy needs further studying.	[73]

Abbreviations: AMP, ampicillin; AMR, antimicrobial resistant bacteria; AST, antimicrobial susceptibility test; ATCC, American Type Culture Collection; CFU, colony forming units; CPT, ciprofloxacin; CTX, cefotaxime; DAP, daptomycin; DAP-R, daptomycin-resistant; DAP-SDD, daptomycin susceptible dose dependent; ERT, erythromycin; FIC, fractional inhibitory concentration; LNZ, linezolid; MDR, multidrug resistant; MIC, minimum inhibitory concentration; MIN, minocycline; OD, optical density; PFU, plaque forming units; ST, sequence type; TEI-R, teicoplanin-resistant; TKA, time-kill assay; VRE, vancomycin-resistant Enterococci; VSE, vancomycin-susceptible Enterococci.

**Table 2:** Summary of ex vivo model assays evaluating phage therapy in *E. faecium*.

No.	Phage(s)	Ex Vivo Model	Objective	Key Findings	Safety Assessment	Observed Limitations	Ref.
1	Max	3T3	Evaluate the lytic efficacy of the phage against *E. faecalis* on epithelial cells.	3 log CFU/mL reduction at 6 h of treatment. 1 log CFU/mL increase in 3T3 cell viability at 6 h.	No cytotoxicity observed at 10^7^ and 10^8^ PFU/mL.	No specific limitations reported in sources.	[48]
2	Zip	3T3	Evaluate the lytic efficacy of the phage against *E. faecium* on epithelial cells.	3 log CFU/mL reduction at 6 h of treatment. Increased 3T3 cell viability at 6 h.	Cytotoxicity observed at 10^8^ PFU/mL in lysate but not in PEG purified phage.	No specific limitations reported in sources.	[48]
3	Max	CWM	Assess the lytic efficacy of the phage against *E. faecalis* biofilms in a wound-simulating model.	2 log CFU/mL reduction at 3 h of treatment. Resistance observed after 24 h.	Not evaluated.	Closed system, lacking immune cells and physiological factors (hypoxia, exudate).	[48]
4	Zip	CWM	Assess the lytic efficacy of the phage against *E. faecium* biofilms in a wound-simulating model.	1.5 log CFU/mL reduction between 3 h and 6 h of treatment. Resistance observed after 24 h.	Not evaluated.	Closed system, lacking immune cells and physiological factors (hypoxia, exudate).	[48]
5	Max and Zip (Cocktail)	CWM	Assess the lytic efficacy of the phage cocktail against multispecies biofilms (*E. faecalis* and *E. faecium*) in a wound-simulating model.	2.5 log CFU/mL reduction at 3 h of treatment. Resistance observed after 24 h.	Not evaluated.	Closed system, lacking immune cells and physiological factors (hypoxia, exudate).	[48]
6	NV-497	SEV	Evaluate the efficacy of the phage in combination with antibiotics against *E. faecium* in a simulated endocardial vegetation model.	Bactericidal activity in combination with DAP, CPT, and NV-497-NV-503-01 cocktail.	Not evaluated.	Model duration of 96 h may not reflect the prolonged treatment needed for infectious endocarditis.	[85]
7	NV-503-01	SEV	Evaluate the efficacy of the phage in combination with antibiotics against *E. faecium* in a simulated endocardial vegetation model.	Bactericidal activity in combination with DAP, CPT, and NV-497-NV-503-01 cocktail.	Not evaluated.	Model duration of 96 h may not reflect the prolonged treatment needed for infectious endocarditis.	[85]
8	NV-497 and NV-503-01 (Cocktail)	SEV	Evaluate the efficacy of the phage cocktail in combination with antibiotics against *E. faecium* in a simulated endocardial vegetation model.	5.77 log CFU/g reduction in combination with DAP and CPT. Re-sensitization to DAP. Prevention of phage resistance in presence of DAP-CPT.	Not evaluated.	Model duration of 96 h; only a single *E. faecium* strain tested, and no whole genome sequencing performed.	[69]

Abbreviations: CWM, collagen wound model; 3T3, epithelial cell colonization model; SEV, simulated endocardial vegetation; CFU, colony forming units; PFU, plaque forming units; DAP, daptomycin; CPT, ceftaroline.

**Table 3:** Summary of in vivo model assays evaluating phage therapy in *E. faecium*.

No.	Phage(s)	In Vivo Model	Objective	Experimental Procedure	Key Findings	Observed Limitations	Ref.
1	MDA1	*G. mellonella*	Evaluate individual phage efficacy and safety against VRE.	Larvae were injected with VRE isolated from a patient. Then, 10^6^ PFU of phage MDA1 was injected 1 h before (preventive) or 1 h after infection (treatment). Survival and health scored at 8 h, 37 °C.	Phage MDA1 significantly increased survival in VRE-infected larvae (80% survival with phage vs. 32% without).	*G. mellonella* immune complexity is limited compared to mammals.	[86]
2	MDA1, MDA2, MDA3, MDA4 (cocktail)	*G. mellonella*	Evaluate the enhanced efficacy and safety of a phage cocktail against VRE.	Larvae injected with VRE isolate followed by 10^6^ PFU of MDA1-MDA4 cocktail at 1 h pre- or post-infection. Health scores and survival tracked at 8 h, 37 °C.	Cocktail improved survival rate to 82%, higher than single DA1 phage (66%); injection with phages alone showed no adverse effects, with control-level survival.	Immune response in *G. mellonella* does not fully represent mammalian systems.	[86]
3	MDA1 and MDA2 (cocktail)	*G. mellonella*	Evaluate the efficacy and safety of a phage cocktail against VRE.	Larvae were injected with VRE, followed by a phage cocktail injection (MDA1 and MDA2) either 1 h pre- or post-infection. Survival monitored over 48 h.	Phage cocktail significantly increased larval survival, and bacterial abundance decreased in treated larvae.	*Galleria* model lacks mammalian immune system complexity.	[53]
4	vB_EfaH_163	*G. mellonella*	Evaluate phage ability to control VRE *E. faecium* in vivo.	Larvae injected with 10^6^ CFU *E. faecium* VR-13, followed by vB_EfaH_163 phage (MOI 0.1) or control (water) 1 h later. Survival monitored over 5 days.	Phage treatment increased survival by 20% compared to control, though not statistically significant.	Small sample size (N = 10) may have affected statistical power.	[73]
5	C33	Mouse (bacteremia)	Demonstrate that lytic activity in vitro is essential for phage rescue in bacteremia.	Bacteremia induced with 3 × 10^9^ CFU *E. faecalis* CRMEN 19. After 90 min, 9 × 10^9^ PFU of either non-lytic ENB6 or lytic C33 were administered i.p., and survival monitored over 20 days.	C33 (lytic) rescued 100% of mice, while non-lytic ENB6 showed no efficacy, underscoring the necessity of lytic activity.	Only two phages and one strain were tested, limiting broader conclusions.	[37]
6	ENB6	Mouse (bacteremia)	Evaluate phage efficacy in rescuing VRE bacteremia mice.	Bacteremia induced by i.p. injection of 10^9^ CFU *E. faecium* CRMEN 44. Then, 45 min post-infection, various doses of ENB6 phage (3 × 10^9^ to 3 × 10^4^ PFU) were administered i.p., and health was monitored over 20 days.	Dose-dependent survival observed; 100% survival at highest doses (MOI 0.3 and 3). Phage treatment reduced blood bacterial load by ~200-fold vs. control.	Focus on one phage and specific strain limits broader applicability.	[37]
7	EF-M80	Mouse (skin wound infection)	Assess hydrogel-encapsulated phage effectiveness in *E. faecium* biofilm-induced wound infections.	Wounds were infected with *E. faecium*, then treated with hydrogel-encapsulated phage 24 h post-infection. Wound healing was monitored for 14 days.	Ph-hyd treatment led to full wound closure by day 14, with initial healing by day 3. Ph-hyd showed faster healing, lower neutrophils, and higher fibroblast density vs. controls.	Limited to a single phage concentration, treatment timing, and mouse model.	[57]

Abbreviations: B, positive infection control; CFU, colony-forming units; *G. mellonella*, *Galleria mellonella*; HC, healthy control; i.p., intraperitoneal; MOI, multiplicity of infection; NC, negative control; PFU, plaque-forming units; Ph-hyd, phage encapsulated in hydrogel; VRE, vancomycin-resistant *Enterococcus*.

**Table 4:** Summary of in vivo human compassionate cases evaluating phage therapy in *E. faecium*.

Nº	Phage(s)	Infection	Administration Route	Treatment Dosage and Duration	Antibiotics Used	Outcome	Ref.
1	EFgrKN, EFgrNG (cocktail)	VRE in pediatric transplant patient with refractory infection.	Intravenous.	2 mL/kg BW, 8.1 × 10^7^ PFU/mL for 10 days, then 5.2 × 10^8^ PFU/mL for 10 days (20 days total), twice daily.	CIP, ERT, CTV, DAP, TIG, CTX, PIT, VAN, LIN, and MER.	Clinical improvement after phage therapy. Treatment was not associated with any adverse events.	[91,94]
2	Φ9184, ΦHi3 (cocktail)	Recurrent VRE and VSE bacteremia in a 57-year-old woman.	Intravenous, oral.	Φ9184 at 1 × 10^9^ PFU/dose, added ΦHi3 at 2 × 10^9^ PFU/dose; 121 days with Φ9184, 95 days with both phages (216 days total).	DAP, VAN, CTL, ORI, TIG, TED, and DOX.	Fewer hospitalizations, improved quality of life; patient passed away from pneumonia 7.5 months post-treatment.	[92]
3	Enf1	Prosthetic infection after aortic arch replacement in 52-year-old patient.	Local application via drainage, intraoperatively, and orally.	1 × 10^8^ PFU/mL for 2 days.	CEP, DAP, LIN, and TOB.	*E. faecium* eradicated; patient died 2 months after phage therapy due to infection by other bacteria.	[93]

Abbreviations: BW, body weight; CEP, cefepime; CIP, ciprofloxacin; CTV, ceftazidime/avibactam; CTL, ceftaroline; CTX, cotrimoxazole; DAP, daptomycin; DOX, doxycycline; ERT, ertapenem; LIN, linezolid; MER, meropenem; MOI, multiplicity of infection; ORI, oritavancin; PFU, plaque-forming units; PIT, piperacillin/tazobactam; TED, tedizolid; TIG, tigecycline; TOB, tobramycin; VAN, vancomycin; VRE, vancomycin-resistant *Enterococcus*; VSE, vancomycin-sensitive *Enterococcus*.

## References

[1] Sanderson H., Gray K.L., Manuele A., Maguire F., Khan A., Liu C., Rudrappa C.N., Nash J.H.E., Robertson J., Bessonov K. (2022). Exploring the Mobilome and Resistome of *Enterococcus faecium* in a One Health Context across Two Continents. Microb. Genom..

[2] Krawczyk B., Wityk P., Gałęcka M., Michalik M. (2021). The Many Faces of *Enterococcus* spp.—Commensal, Probiotic and Opportunistic Pathogen. Microorganisms.

[3] Hanchi H., Mottawea W., Sebei K., Hammami R. (2018). The Genus *Enterococcus*: Between Probiotic Potential and Safety Concerns-an Update. Front. Microbiol..

[4] Gao W., Howden B.P., Stinear T.P. (2018). Evolution of Virulence in *Enterococcus faecium*, a Hospital-Adapted Opportunistic Pathogen. Curr. Opin. Microbiol..

[5] Horner C., Mushtaq S., Allen M., Hope R., Gerver S., Longshaw C., Reynolds R., Woodford N., Livermore D.M. (2021). Replacement of *Enterococcus faecalis* by *Enterococcus faecium* as the Predominant *Enterococcus* in UK Bacteraemias. JAC-Antimicrob. Resist..

[6] Zhou X., Willems R.J.L., Friedrich A.W., Rossen J.W.A., Bathoorn E. (2020). *Enterococcus faecium*: From Microbiological Insights to Practical Recommendations for Infection Control and Diagnostics. Antimicrob. Resist. Infect. Control.

[7] Uda A., Shigemura K., Kitagawa K., Osawa K., Onuma K., Yan Y., Nishioka T., Fujisawa M., Yano I., Miyara T. (2021). Risk Factors for the Acquisition of *Enterococcus faecium* Infection and Mortality in Patients with Enterococcal Bacteremia: A 5-Year Retrospective Analysis in a Tertiary Care University Hospital. Antibiotics.

[8] Hornuss D., Göpel S., Walker S.V., Tobys D., Häcker G., Seifert H., Higgins P.G., Xanthopoulou K., Gladstone B.P., Cattaneo C. (2024). Epidemiological Trends and Susceptibility Patterns of Bloodstream Infections Caused by *Enterococcus* spp. in Six German University Hospitals: A Prospectively Evaluated Multicentre Cohort Study from 2016 to 2020 of the R-Net Study Group. Infection.

[9] Lupia T., Roberto G., Scaglione L., Shbaklo N., De benedetto I., Scabini S., Mornese Pinna S., Curtoni A., Cavallo R., De Rosa F.G. (2022). Clinical and Microbiological Characteristics of Bloodstream Infections Caused by *Enterococcus* spp. within Internal Medicine Wards: A Two-Year Single-Centre Experience. Intern. Emerg. Med..

[10] Moemen D., Tawfeek D., Badawy W. (2015). Healthcare-Associated Vancomycin Resistant *Enterococcus faecium* Infections in the Mansoura University Hospitals Intensive Care Units, Egypt. Braz. J. Microbiol..

[11] van Hal S.J., Willems R.J.L., Gouliouris T., Ballard S.A., Coque T.M., Hammerum A.M., Hegstad K., Westh H.T., Howden B.P., Malhotra-Kumar S. (2021). The Global Dissemination of Hospital Clones of *Enterococcus faecium*. Genome Med..

[12] Brinkwirth S., Ayobami O., Eckmanns T., Markwart R. (2021). Hospital-Acquired Infections Caused by Enterococci: A Systematic Review and Meta-Analysis, Who European Region, 1 January 2010 to 4 February 2020. Eurosurveillance.

[13] Levitus M., Rewane A., Perera T.B. (2023). Vancomycin-Resistant Enterococci.

[14] Arias C.A., Murray B.E. (2012). The Rise of the *Enterococcus*: Beyond Vancomycin Resistance. Nat. Rev. Microbiol..

[15] Hollenbeck B.L., Rice L.B. (2012). Intrinsic and Acquired Resistance Mechanisms in *Enterococcus*. Virulence.

[16] Cimen C., Berends M.S., Bathoorn E., Lokate M., Voss A., Friedrich A.W., Glasner C., Hamprecht A. (2023). Vancomycin-Resistant Enterococci (VRE) in Hospital Settings across European Borders: A Scoping Review Comparing the Epidemiology in the Netherlands and Germany. Antimicrob. Resist. Infect. Control.

[17] Sugai M., Yuasa A., Miller R.L., Vasilopoulos V., Kurosu H., Taie A., Gordon J.P., Matsumoto T. (2023). Correction to: An Economic Evaluation Estimating the Clinical and Economic Burden of Increased Vancomycin-Resistant *Enterococcus faecium* Infection Incidence in Japan. Infect. Dis. Ther..

[18] Wei Y., Palacios Araya D., Palmer K.L. (2024). *Enterococcus faecium*: Evolution, Adaptation, Pathogenesis and Emerging Therapeutics. Nat. Rev. Microbiol..

[19] Blane B., Coll F., Raven K., Allen O., Kappeler A.R.M., Pai S., Floto R.A., Peacock S.J., Gouliouris T. (2023). Impact of a New Hospital with Close to 100% Single-Occupancy Rooms on Environmental Contamination and Incidence of Vancomycin-Resistant *Enterococcus faecium* Colonization or Infection: A Genomic Surveillance Study. J. Hosp. Infect..

[20] Kristich C.J., Rice L.B., Arias C.A. (2014). Enterococcal Infection—Treatment and Antibiotic Resistance. Enterococci from Commensals to Leading Causes of Drug Resistant Infection.

[21] Sattari-Maraji A., Jabalameli F., Node Farahani N., Beigverdi R., Emaneini M. (2019). Antimicrobial Resistance Pattern, Virulence Determinants and Molecular Analysis of *Enterococcus Faecium* Isolated from Children Infections in Iran. BMC Microbiol..

[22] Wei M., Wang P., Li T., Wang Q., Su M., Gu L., Wang S. (2023). Antimicrobial and Antibiofilm Effects of Essential Fatty Acids against Clinically Isolated Vancomycin-Resistant *Enterococcus faecium*. Front. Cell. Infect. Microbiol..

[23] Șchiopu P., Toc D.A., Colosi I.A., Costache C., Ruospo G., Berar G., Gălbău Ș.-G., Ghilea A.C., Botan A., Pană A.G. (2023). An Overview of the Factors Involved in Biofilm Production by the *Enterococcus* Genus. Int. J. Mol. Sci..

[24] Miller W.R., Arias C.A. (2024). ESKAPE Pathogens: Antimicrobial Resistance, Epidemiology, Clinical Impact and Therapeutics. Nat. Rev. Microbiol..

[25] Lin D.M., Koskella B., Lin H.C. (2017). Phage Therapy: An Alternative to Antibiotics in the Age of Multi-Drug Resistance. World J. Gastrointest. Pharmacol. Ther..

[26] Chung K.M., Liau X.L., Tang S.S. (2023). Bacteriophages and Their Host Range in Multidrug-Resistant Bacterial Disease Treatment. Pharmaceuticals.

[27] Sahu R., Singh A.K., Kumar A., Singh K., Kumar P. (2023). Bacteriophages Concept and Applications: A Review on Phage Therapy. Curr. Pharm. Biotechnol..

[28] Sulakvelidze A., Alavidze Z., Morris J. (2001). Bacteriophage Therapy. Antimicrob. Agents Chemother..

[29] Brives C., Pourraz J. (2020). Phage Therapy as a Potential Solution in the Fight against AMR: Obstacles and Possible Futures. Palgrave Commun..

[30] Summers W.C. (2012). The Strange History of Phage Therapy. Bacteriophage.

[31] Walter N., Mirzaei M.K., Deng L., Willy C., Alt V., Rupp M. (2024). The Potential of Bacteriophage Therapy as an Alternative Treatment Approach for Antibiotic-Resistant Infections. Med. Princ. Pract..

[32] Fortaleza J.A.G., Ong C.J.N., De Jesus R. (2024). Efficacy and Clinical Potential of Phage Therapy in Treating Methicillin-Resistant *Staphylococcus aureus* (MRSA) Infections: A Review. Eur. J. Microbiol. Immunol..

[33] Eskenazi A., Lood C., Wubbolts J., Hites M., Balarjishvili N., Leshkasheli L., Askilashvili L., Kvachadze L., van Noort V., Wagemans J. (2022). Combination of Pre-Adapted Bacteriophage Therapy and Antibiotics for Treatment of Fracture-Related Infection Due to Pandrug-Resistant *Klebsiella pneumoniae*. Nat. Commun..

[34] Chegini Z., Khoshbayan A., Taati Moghadam M., Farahani I., Jazireian P., Shariati A. (2020). Bacteriophage Therapy against *Pseudomonas aeruginosa* Biofilms: A Review. Ann. Clin. Microbiol. Antimicrob..

[35] Broncano-Lavado A., Santamaría-Corral G., Esteban J., García-Quintanilla M. (2021). Advances in Bacteriophage Therapy against Relevant Multidrug-Resistant Pathogens. Antibiotics.

[36] McCallin S., Sacher J.C., Zheng J., Chan B.K. (2019). Current State of Compassionate Phage Therapy. Viruses.

[37] Biswas B., Adhya S., Washart P., Paul B., Trostel A.N., Powell B., Carlton R., Merril C.R. (2002). Bacteriophage Therapy Rescues Mice Bacteremic from a Clinical Isolate of Vancomycin-Resistant *Enterococcus faecium*. Infect. Immun..

[38] Zhu X., Tang L., Wang Z., Xie F., Zhang W., Li Y. (2024). A Comparative Analysis of Phage Classification Methods in Light of the Recent ICTV Taxonomic Revisions. Virology.

[39] Turner D., Kropinski A.M., Adriaenssens E.M. (2021). A Roadmap for Genome-Based Phage Taxonomy. Viruses.

[40] Gong P., Cheng M., Li X., Jiang H., Yu C., Kahaer N., Li J., Zhang L., Xia F., Hu L. (2016). Characterization of *Enterococcus faecium* Bacteriophage IME-EFm5 and Its Endolysin LysEFm5. Virology.

[41] Rigvava S., Kusradze I., Tchgkonia I., Karumidze N., Dvalidze T., Goderdzishvili M. (2022). Novel Lytic Bacteriophage VB_GEC_EfS_9 against *Enterococcus Faecium*. Virus Res..

[42] Wang Y., Wang W., Lv Y., Zheng W., Mi Z., Pei G., An X., Xu X., Han C., Liu J. (2014). Characterization and Complete Genome Sequence Analysis of Novel Bacteriophage IME-EFm1 Infecting *Enterococcus faecium*. J. Gen. Virol..

[43] Goodarzi F., Hallajzadeh M., Sholeh M., Talebi M., Pirhajati Mahabadi V., Amirmozafari N. (2021). Biological Characteristics and Anti-Biofilm Activity of a Lytic Phage against Vancomycin-Resistant *Enterococcus faecium*. Iran. J. Microbiol..

[44] Canfield G.S., Chatterjee A., Espinosa J., Mangalea M.R., Sheriff E.K., Keidan M., McBride S.W., McCollister B.D., Hang H.C., Duerkop B.A. (2021). Lytic Bacteriophages Facilitate Antibiotic Sensitization of *Enterococcus faecium*. Antimicrob. Agents Chemother..

[45] Lee D., Im J., Na H., Ryu S., Yun C.H., Han S.H. (2019). The Novel *Enterococcus* Phage VB_EfaS_HEf13 Has Broad Lytic Activity Against Clinical Isolates of *Enterococcus faecalis*. Front. Microbiol..

[46] Topka-Bielecka G., Nejman-Faleńczyk B., Bloch S., Dydecka A., Necel A., Węgrzyn A., Węgrzyn G. (2021). Phage–Bacteria Interactions in Potential Applications of Bacteriophage Vb_efas-271 against *Enterococcus faecalis*. Viruses.

[47] Tkachev P.V., Pchelin I.M., Azarov D.V., Gorshkov A.N., Shamova O.V., Dmitriev A.V., Goncharov A.E. (2022). Two Novel Lytic Bacteriophages Infecting *Enterococcus* spp. Are Promising Candidates for Targeted Antibacterial Therapy. Viruses.

[48] Melo L.D.R., Ferreira R., Costa A.R., Oliveira H., Azeredo J. (2019). Efficacy and Safety Assessment of Two Enterococci Phages in an in Vitro Biofilm Wound Model. Sci. Rep..

[49] Nacharov P.V., Krivopalov A.A., Shustova T.I. (2023). General Characteristics, Results and Prospects for the Clinical Application of Bacteriophage Therapy. Meditsinskiy Sov. = Med. Counc..

[50] Friedrich L., Curran M., Chitra S., Manley A., Bai S., Noble B., Steenbergen J.N., Garrity-Ryan L., Tzanis E. (2019). 734. Modeling the Pharmacokinetics and Pharmacodynamics of Intravenous and Oral Omadacycline With and Without a Loading Dose.

[51] Wandro S., Oliver A., Gallagher T., Weihe C., England W., Martiny J.B.H., Whiteson K. (2019). Predictable Molecular Adaptation of Coevolving *Enterococcus faecium* and Lytic Phage EfV12-Phi1. Front. Microbiol..

[52] Jarvis A.W., Collins L.J., Ackermann H.W. (1993). A Study of Five Bacteriophages of the Myoviridae Family Which Replicate on Different Gram-Positive Bacteria. Arch. Virol..

[53] El Haddad L., Angelidakis G., Clark J.R., Mendoza J.F., Terwilliger A.L., Chaftari C.P., Duna M., Yusuf S.T., Harb C.P., Stibich M. (2022). Genomic and Functional Characterization of Vancomycin-Resistant Enterococci-Specific Bacteriophages in the *Galleria mellonella* Wax Moth Larvae Model. Pharmaceutics.

[54] Lossouarn J., Beurrier E., Bouteau A., Moncaut E., Sir Silmane M., Portalier H., Zouari A., Cattoir V., Serror P., Petit M.-A.A. (2024). The Virtue of Training: Extending Phage Host Spectra against Vancomycin-Resistant *Enterococcus Faecium* Strains Using the Appelmans Method. Antimicrob. Agents Chemother..

[55] Buzikov R.M., Kazantseva O.A., Piligrimova E.G., Ryabova N.A., Shadrin A.M. (2023). Bacteriolytic Potential of *Enterococcus* Phage IF6 Isolated from “Sextaphag^®^” Therapeutic Phage Cocktail and Properties of Its Endolysins, Gp82 and Gp84. Viruses.

[56] Wang Z., Fokine A., Guo X., Jiang W., Rossmann M.G., Kuhn R.J., Luo Z.-H., Klose T. (2023). Structure of Vibrio Phage XM1, a Simple Contractile DNA Injection Machine. Viruses.

[57] Khazani Asforooshani M., Elikaei A., Abed S., Shafiei M., Barzi S.M., Solgi H., Badmasti F., Sohrabi A. (2024). A Novel *Enterococcus faecium* Phage EF-M80: Unveiling the Effects of Hydrogel-Encapsulated Phage on Wound Infection Healing. Front. Microbiol..

[58] Soro O., Kigen C., Nyerere A., Gachoya M., Georges M., Odoyo E., Musila L. (2024). Characterization and Anti-Biofilm Activity of Lytic *Enterococcus* Phage VB_Efs8_KEN04 against Clinical Isolates of Multidrug-Resistant *Enterococcus faecalis* in Kenya. Viruses.

[59] Baharuddin A., Abdelkarim A., Marghani E., Ahmed I.A., Samian M.R. (2017). Revitalizing Phage Therapy in Combating Multi-Drug Resistant Bacteria. Haya Saudi J. Life Sci..

[60] Thomas L.V., Ockhuizen T., Suzuki K. (2014). Exploring the Influence of the Gut Microbiota and Probiotics on Health: A Symposium Report. Br. J. Nutr..

[61] Górska A., Przystupski D., Niemczura M.J., Kulbacka J. (2019). Probiotic Bacteria: A Promising Tool in Cancer Prevention and Therapy. Curr. Microbiol..

[62] Federici S., Kviatcovsky D., Valdés-Mas R., Elinav E. (2023). Microbiome-Phage Interactions in Inflammatory Bowel Disease. Clin. Microbiol. Infect..

[63] Raza T., Andleeb S., Ullah S.R., Jamal M., Mehmood K., Ali M. (2018). Isolation and Characterization of a Phage to Control Vancomycin Resistant *Enterococcus faecium*. Open Life Sci..

[64] Qu Q., Chen T., He P., Geng H., Zeng P., Luan G. (2023). Isolation and Characterization of a Novel Lytic Bacteriophage VB_Efm_LG62 Infecting *Enterococcus faecium*. Virus Genes.

[65] Lorenzo-Rebenaque L., Casto-Rebollo C., Diretto G., Frusciante S., Rodríguez J.C., Ventero M.-P., Molina-Pardines C., Vega S., Marin C., Marco-Jiménez F. (2022). Examining the Effects of Salmonella Phage on the Caecal Microbiota and Metabolome Features in Salmonella-Free Broilers. Front. Genet..

[66] Fernández L., Gutiérrez D., García P., Rodríguez A. (2019). The Perfect Bacteriophage for Therapeutic Applications—A Quick Guide. Antibiotics.

[67] Abedon S.T. (2011). Size Does Matter—Distinguishing Bacteriophages by Genome Length (and ‘Breadth’). Microbiol. Aust..

[68] Li J., Shi H., Zhao C., Hao Y., He Y., Sun Y. (2016). Complete Genome Sequence of the Siphoviral Bacteriophage Ec-ZZ2, Which Is Capable of Lysing *Enterococcus faecium*. Genome Announc..

[69] Soro O., Kigen C., Nyerere A., Gachoya M., Wachira J., Onyonyi V., Georges M., Wataka A., Ondolo S., Cherono K. (2024). Complete Genome Sequences of Four Lytic Bacteriophages against Multidrug-Resistant *Enterococcus faecium*. Microbiol. Resour. Announc..

[70] Altamirano F.L.G., Barr J.J. (2021). Screening for Lysogen Activity in Therapeutically Relevant Bacteriophages. Bio-Protocol.

[71] Beamud B., Benz F., Bikard D. (2024). Going Viral: The Role of Mobile Genetic Elements in Bacterial Immunity. Cell Host Microbe.

[72] Mayo-Muñoz D., Pinilla-Redondo R., Camara-Wilpert S., Birkholz N., Fineran P.C. (2024). Inhibitors of Bacterial Immune Systems: Discovery, Mechanisms and Applications. Nat. Rev. Genet..

[73] Pradal I., Casado A., del Rio B., Rodriguez-Lucas C., Fernandez M., Alvarez M.A., Ladero V. (2023). *Enterococcus Faecium* Bacteriophage VB_EfaH_163, a New Member of the Herelleviridae Family, Reduces the Mortality Associated with an *E. faecium* VanR Clinical Isolate in a *Galleria mellonella* Animal Model. Viruses.

[74] Lev K., Coyne A.J.K., Kebriaei R., Morrisette T., Stamper K., Holger D.J., Canfield G.S., Duerkop B.A., Arias C.A., Rybak M.J. (2022). Evaluation of Bacteriophage-Antibiotic Combination Therapy for Biofilm-Embedded MDR *Enterococcus Faecium*. Antibiotics.

[75] Coyne A.J.K., Stamper K., Kebriaei R., Holger D.J., El Ghali A., Morrisette T., Biswas B., Wilson M., Deschenes M.V., Canfield G.S. (2022). Phage Cocktails with Daptomycin and Ampicillin Eradicates Biofilm-Embedded Multidrug-Resistant *Enterococcus faecium* with Preserved Phage Susceptibility. Antibiotics.

[76] Kunz Coyne A.J., Eshaya M., Bleick C., Vader S., Biswas B., Wilson M., Deschenes M.V., Alexander J., Lehman S.M., Rybak M.J. (2024). Exploring Synergistic and Antagonistic Interactions in Phage-Antibiotic Combinations against ESKAPE Pathogens. Microbiol. Spectr..

[77] Morrisette T., Lev K.L., Kebriaei R., Abdul-Mutakabbir J.C., Stamper K.C., Morales S., Lehman S.M., Canfield G.S., Duerkop B.A., Arias C.A. (2020). Bacteriophage-Antibiotic Combinations for *Enterococcus faecium* with Varying Bacteriophage and Daptomycin Susceptibilities. Antimicrob. Agents Chemother..

[78] Morrisette T., Lev K.L., Canfield G.S., Duerkop B.A., Kebriaei R., Stamper K.C., Holger D., Lehman S.M., Willcox S., Arias C.A. (2022). Evaluation of Bacteriophage Cocktails Alone and in Combination with Daptomycin against Daptomycin-Nonsusceptible *Enterococcus faecium*. Antimicrob. Agents Chemother..

[79] Wandro S., Ghatbale P., Attai H., Hendrickson C., Samillano C., Suh J., Dunham S.J.B., Pride D.T., Whiteson K. (2022). Phage Cocktails Constrain the Growth of *Enterococcus*. mSystems.

[80] Yoong P., Schuch R., Nelson D., Fischetti V.A. (2004). Identification of a Broadly Active Phage Lytic Enzyme with Lethal Activity against Antibiotic-Resistant *Enterococcus faecalis* and *Enterococcus faecium*. J. Bacteriol..

[81] Gu Liu C., Green S.I., Min L., Clark J.R., Salazar K.C., Terwilliger A.L., Kaplan H.B., Trautner B.W., Ramig R.F., Maresso A.W. (2020). Phage-Antibiotic Synergy Is Driven by a Unique Combination of Antibacterial Mechanism of Action and Stoichiometry. mBio.

[82] Fungo G.B.N., Uy J.C.W., Porciuncula K.L.J., Candelario C.M.A., Chua D.P.S., Gutierrez T.A.D., Clokie M.R.J., Papa D.M.D. (2023). “Two Is Better Than One”: The Multifactorial Nature of Phage-Antibiotic Combinatorial Treatments Against ESKAPE-Induced Infections. PHAGE.

[83] Łusiak-Szelachowska M., Międzybrodzki R., Drulis-Kawa Z., Cater K., Knežević P., Winogradow C., Amaro K., Jończyk-Matysiak E., Weber-Dąbrowska B., Rękas J. (2022). Bacteriophages and Antibiotic Interactions in Clinical Practice: What We Have Learned so Far. J. Biomed. Sci..

[84] Gurney J., Brown S.P., Kaltz O., Hochberg M.E. (2020). Steering Phages to Combat Bacterial Pathogens. Trends Microbiol..

[85] Kunz Coyne A.J., Stamper K., El Ghali A., Kebriaei R., Biswas B., Wilson M., Deschenes M.V., Tran T.T., Arias C.A., Rybak M.J. (2023). Phage-Antibiotic Cocktail Rescues Daptomycin and Phage Susceptibility against Daptomycin-Nonsusceptible *Enterococcus faecium* in a Simulated Endocardial Vegetation Ex Vivo Model. Microbiol. Spectr..

[86] El Haddad L., Harb C.P., Stibich M., Chemaly R.F., Chemaly R.F. (2019). 735. Bacteriophage Therapy Improves Survival of *Galleria mellonella* Larvae Injected with Vancomycin-Resistant *Enterococcus faecium*. Open Forum Infect. Dis..

[87] Loh J.M., Adenwalla N., Wiles S., Proft T. (2013). *Galleria mellonella* Larvae as an Infection Model for Group A Streptococcus. Virulence.

[88] Desbois A.P., Coote P.J. (2012). Utility of Greater Wax Moth Larva (*Galleria mellonella*) for Evaluating the Toxicity and Efficacy of New Antimicrobial Agents. Adv. Appl. Microbiol..

[89] Champion O.L., Wagley S., Titball R.W. (2016). *Galleria mellonella* as a Model Host for Microbiological and Toxin Research. Virulence.

[90] Geier M.R., Trigg M.E., Merril C.R. (1973). Fate of Bacteriophage Lambda in Non-Immune Germ-Free Mice. Nature.

[91] Paul K., Merabishvili M., Hazan R., Christner M., Herden U., Gelman D., Khalifa L., Yerushalmy O., Coppenhagen-Glazer S., Harbauer T. (2021). Bacteriophage Rescue Therapy of a Vancomycin-Resistant *Enterococcus faecium* Infection in a One-Year-Old Child Following a Third Liver Transplantation. Viruses.

[92] Stellfox M.E., Fernandes C., Shields R.K., Haidar G., Kramer K.H., Dembinski E., Mangalea M.R., Arya G., Canfield G.S., Duerkop B.A. (2024). Bacteriophage and Antibiotic Combination Therapy for Recurrent *Enterococcus faecium* Bacteremia. mBio.

[93] Rubalskii E., Ruemke S., Salmoukas C., Boyle E.C., Warnecke G., Tudorache I., Shrestha M., Schmitto J.D., Martens A., Rojas S.V. (2020). Bacteriophage Therapy for Critical Infections Related to Cardiothoracic Surgery. Antibiotics.

[94] Pirnay J.P., Djebara S., Steurs G., Griselain J., Cochez C., De Soir S., Glonti T., Spiessens A., Vanden Berghe E., Green S. (2024). Personalized Bacteriophage Therapy Outcomes for 100 Consecutive Cases: A Multicentre, Multinational, Retrospective Observational Study. Nat. Microbiol..

[95] Pires D.P., Costa A.R., Pinto G., Meneses L., Azeredo J. (2020). Current Challenges and Future Opportunities of Phage Therapy. FEMS Microbiol. Rev..

[96] Chan B.K., Sistrom M., Wertz J.E., Kortright K.E., Narayan D., Turner P.E. (2016). Phage Selection Restores Antibiotic Sensitivity in MDR *Pseudomonas aeruginosa*. Sci. Rep..

[97] Suh G.A., Lodise T.P., Tamma P.D., Knisely J.M., Alexander J., Aslam S., Barton K.D., Bizzell E., Totten K.M.C., Campbell J.L. (2022). Considerations for the Use of Phage Therapy in Clinical Practice. Antimicrob. Agents Chemother..

[98] Luong T., Salabarria A.-C., Roach D.R. (2020). Phage Therapy in the Resistance Era: Where Do We Stand and Where Are We Going?. Clin. Ther..

